# Anti-Angiogenetic and Anti-Lymphangiogenic Effects of a Novel 2-Aminobenzimidazole Derivative, MFB

**DOI:** 10.3389/fonc.2022.862326

**Published:** 2022-06-20

**Authors:** Ming-Jen Hsu, Han-Kun Chen, Cheng-Yu Chen, Jin-Cherng Lien, Jing-Yan Gao, Yu-Han Huang, Justin Bo-Kai Hsu, Gilbert Aaron Lee, Shiu-Wen Huang

**Affiliations:** ^1^ Graduate Institute of Medical Sciences, College of Medicine, Taipei Medical University, Taipei, Taiwan; ^2^ Department of Pharmacology, School of Medicine, College of Medicine, Taipei Medical University, Taipei, Taiwan; ^3^ Cell Physiology and Molecular Image Research Center, Wan Fang Hospital, Taipei Medical University, Taipei, Taiwan; ^4^ Department of General Surgery, Chi Mei Medical Center, Tainan, Taiwan; ^5^ Translational Imaging Research Center, Taipei Medical University Hospital, Taipei, Taiwan; ^6^ Department of Radiology, National Defense Medical Center, Taipei, Taiwan; ^7^ Research Center for Artificial Intelligence in Medicine, Taipei Medical University, Taipei, Taiwan; ^8^ Department of Medical Imaging, Taipei Medical University Hospital, Taipei, Taiwan; ^9^ Department of Radiology, School of Medicine, College of Medicine, Taipei Medical University, Taipei, Taiwan; ^10^ School of Pharmacy, China Medical University, Taichung, Taiwan; ^11^ Department of Medical Research, Hospital of China Medical University, Taichung, Taiwan; ^12^ Division of Genetics and Genomics, Department of Pediatrics, Boston Children’s Hospital and Harvard Medical School, Boston, MA, United States; ^13^ The Manton Center for Orphan Disease Research, Boston Children’s Hospital, Boston, MA, United States; ^14^ Department of Medical Research; Research Center of Thoracic Medicine, Taipei Medical University Hospital, Taipei, Taiwan; ^15^ Department of Microbiology and Immunology, School of Medicine, College of Medicine, Taipei Medical University, Taipei, Taiwan; ^16^ Research Center of Thoracic Medicine, Taipei Medical University Hospital, Taipei, Taiwan

**Keywords:** angiogenesis, aminobenzimidazole, human umbilical vascular endothelial cells (HUVECs), lymphatic endothelial cells (LECs), vascular endothelial growth factor (VEGF)

## Abstract

**Background and Purpose:**

Benzimidazoles have attracted much attention over the last few decades due to their broad-spectrum pharmacological properties. Increasing evidence is showing the potential use of benzimidazoles as anti-angiogenic agents, although the mechanisms that impact angiogenesis remain to be fully defined. In this study, we aim to investigate the anti-angiogenic mechanisms of MFB, a novel 2-aminobenzimidazole derivative, to develop a novel angiogenesis inhibitor.

**Experimental Approach:**

MTT, BrdU, migration and invasion assays, and immunoblotting were employed to examine MFB’s effects on vascular endothelial growth factor (VEGF)-induced endothelial cell proliferation, migration, invasion, as well as signaling molecules activation. The anti-angiogenic effects of MFB were analyzed by tube formation, aorta ring sprouting, and matrigel plug assays. We also used a mouse model of lung metastasis to determine the MFB’s anti-metastatic effects.

**Key Results:**

MFB suppressed cell proliferation, migration, invasion, and endothelial tube formation of VEGF-A-stimulated human umbilical vascular endothelial cells (HUVECs) or VEGF-C-stimulated lymphatic endothelial cells (LECs). MFB suppressed VEGF-A and VEGF-C signaling in HUVECs or LECs. In addition, MFB reduced VEGF-A- or tumor cells-induced neovascularization *in vivo.* MFB also diminished B16F10 melanoma lung metastasis. The molecular docking results further showed that MFB may bind to VEGFR-2 rather than VEGF-A with high affinity.

**Conclusions and Implications:**

These observations indicated that MFB may target VEGF/VEGFR signaling to suppress angiogenesis and lymphangiogenesis. It also supports the role of MFB as a potential lead in developing novel agents for the treatment of angiogenesis- or lymphangiogenesis-associated diseases and cancer.

## Introduction

Angiogenesis and lymphangiogenesis are distinct and complex processes of forming new capillary blood and lymphatic vessels from mature existing ones. It occurs primarily in physiological processes such as embryogenesis, tissue repair, reproduction, or the resolution of inflammatory reactions. However, a variety of pathological states including psoriasis, diabetic retinopathy, inflammatory diseases, or cancer also involve angiogenesis and lymphangiogenesis (1, 2). Based on the annual statistics reported by World Health Organization (WHO), cancer remains a major life burden and continues to grow globally. Tens of millions of people are diagnosed with cancer each year and more than half of these patients eventually die from it. Approximately 90% of cancer-related deaths are caused by metastatic tumor spread (3). Dissemination *via* blood vessels (hematogenous spread) or lymphatic vasculature (lymphogenous spread) is currently being recognized as the major route of cancer cell spread (4). Increased numbers of tumor-associated blood and lymphatic vessels are closely associated with tumor metastasis and poor clinical outcome (5, 6). Tumor angiogenesis and lymphangiogenesis have thus emerged as crucial prognostic factors for cancer patients. They also represent rational and promising therapeutic targets for cancer intervention (7).

Vascular endothelial growth factors (VEGFs) and their cognate receptors are central regulators of angiogenesis and lymphangiogenesis (8). The VEGF family comprises five members, namely VEGF-A, VEGF-B, VEGF-C, VEGF-D, and placental growth factor (PLGF). Among these growth factors, VEGF-A has been identified as the most critical angiogenesis-promoting factor (9). VEGF-A is highly expressed in many known tumors and its expression is associated with poor prognosis in cancer patients (10). VEGF-A-induced angiogenesis is primarily mediated by cell surface receptor tyrosine kinase (RTK) termed VEGF receptor (VEGFR)-2 on vascular endothelial cells (11). VEGF-A binding to VEGFR-2 leads to its phosphorylation and initiates downstream signaling pathways such as Src, FAK, Akt, and ERK that are responsible for orchestrating angiogenesis (12, 13). On the other hand, newly formed capillary lymphatic vessels from pre-existing lymphatic vasculature involve the processes including cell proliferation, migration, and tube formation of lymphatic endothelial cells (LECs) that are thought to be similar to angiogenesis. VEGF-C is currently the best-characterized lymphangiogenic factor. VEGF-C augments lymphangiogenic steps through binding to VEGFR-3 (also known as flt-4), which is expressed largely restricted to LECs (14). As a consequence, VEGFR-3 undergoes phosphorylation, leading to the activation of downstream signaling cascades required for cell proliferation, migration and tube formation of LECs. Therefore, targeting VEGF-A/VEGFR-2 or VEGF-C/VEGFR-3 signaling represents a promising strategy for the treatment of angiogenesis- or lymphangiogenesis-related diseases, particularly for intervention of cancer (7, 15).

Multiple approaches targeting VEGF-A/VEGFR-2 or VEGF-C/VEGFR-3 signaling have been developed and assessed in clinical trials (16). These include neutralizing monoclonal antibodies against VEGF-A, VEGF-C, or VEGFRs (7, 17) and soluble decoy receptors (VEGF-Trap) that sequester VEGF-A and/or VEGF-C (18, 19). Small molecule inhibitors that suppress VEGFR-2 and/or VEGFR-3 kinase activity represent another strategy to suppress VEGF signaling (20, 21). To date, several VEGF/VEGFR-targeting agents have been approved by the European Medicines Agency (EMA) or the U.S. Food and Drug Administration (FDA) or in the development pipeline for the treatment of certain types of cancer. These included monoclonal antibodies bevacizumab (Avastin^®^) and ramucirumab (Cyramzar^®^) (22, 23) and small molecule inhibitors such as sunitinib (Sutent^®^), sorafenib (Nexavar^®^), axitinib (Votrient^®^), pazopanib (Votrient^®^) regorafenib (Stivarga^®^) and lenvatinib (Lenvina^®^) (24–27).

Benzimidazole derivatives have drawn great interest over the last few decades because of their beneficial biological and pharmacological properties such as anti-microbial, anti-viral (28), anti-diabetic (29), anti-inflammatory (30) and anti-tumor (31) activities. Many benzimidazole-based small molecule drugs are currently in clinical use for certain diseases or in clinical development for cancer therapy (32). Although the underlying mechanisms remain incompletely understood, increasing evidence is showing the potential use of benzimidazole derivatives as anti-angiogenic agents (12, 33). It appears that additional novel benzimidazole-based compounds may exhibit pharmacological activities capable of clinical application. Therefore, the discovery and synthesis of novel benzimidazole-based compounds remain a major focus in the drug discovery field. However, much effort has been made to explore the biological activities of 2-arylbenzimidazoles. Only a few studies are focusing on investigating the pharmacological properties of 2-aminobenzimidazoles. Given their potential as a lead for drug discovery, we recently synthesized two novel 2-aminobenzimidazole-containing small molecules, namely MFBre and MFB [1-(4-chlorobenzyl)-2-(5-methyl-2-furfurylideneamino)-benzimidazole], and examined their anti-angiogenic activities. In the present study, we aimed to explore the anti-angiogenic mechanisms of MFB. The effects of MFB on lymphangiogenesis will also be investigated in lymphatic endothelial cells.

## Materials and Methods

### Reagents

MFB, a benzimidazole-based compound, was synthesized as described in the Supporting Information. Other compounds and materials were obtained as follows: TrypLE™, Fetal bovine serum (FBS), Medium 199 (M199), and all cell culture reagents were from Invitrogen (Carlsbad, CA, U.S.A.). Recombinant VEGF-A and VEGF-C were from PeproTech (Rocky Hill, NJ, USA). Sunitinib and sorafenib were obtained from SelleckChem (Houston, TX, U.S.A.). All materials for immunoblotting were obtained from Bio-Rad (Hercules, CA, U.S.A.). Antibodies against ERK1/2 (Cell Signaling Technology Cat# 4695), ERK1/2 phosphorylated at threonine 202/tyrosine 204 (T202/Y204) (Cell Signaling Technology Cat# 4370), Akt (Cell Signaling Technology Cat# 9272), Akt phosphorylated at serine 473 (S473) (Cell Signaling Technology Cat# 9271), FAK (Cell Signaling Technology Cat# 3285), FAK phosphorylated at tyrosine 397 (Y397) (Cell Signaling Technology Cat# 3283), VEGFR-2 (Cell Signaling Technology Cat# 2479), VEGFR-2 phosphorylated at tyrosine 1175 (Y1175) (Cell Signaling Technology Cat# 3770) were purchased from Cell Signaling (Danvers, MA, USA). Antibody against α-tubulin (GeneTex Cat# GTX628802), as well as anti-rabbit and anti-mouse IgG conjugated horseradish peroxidase antibodies were obtained from GeneTex Inc (Irvine, CA, U.S.A.). The enhanced chemiluminescence detection kit was from Millipore (Billerica, MA, U.S.A.). Cell Proliferation ELISA, BrdU assay kit was from Roche (Indianapolis, IN, USA). BD Matrigel™ Basement Membrane Matrix was from Becton Dickinson (Mountain View, CA, USA), Toluidine blue O, 3-[4, 5-dimethylthiazol-2-yl]-2, 5-diphenyltetrazolium bromide (MTT), and all other chemicals were from Sigma-Aldrich (St Louis, MO, U.S.A).

### Synthesis of MFB

MFB [1-(4-chlorobenzyl)-2-(5-methyl-2-furfurylideneamino)-benzimidazole], an aminobenzimidazole-based compound, was synthesized as described in the “**Supplement Information**”. MFB is dissolved in dimethyl sulfoxide (DMSO). The vehicle used in the control group in the absence of MFB is 0.1% DMSO.

### Cell Culture

Human umbilical vascular endothelial cells (HUVECs), GBM8901 (BCRC Cat# 60164) (Chin. Med. J. (Taipei) 48: 177-184, 1991) glioblastoma and B16F10 (BCRC Cat# 60031) melanoma cell lines were obtained from the Bioresource Collection and Research Center (Hsinchu, Taiwan). HUVECs were maintained in M199 medium containing 10% FBS, 20 mM HEPES, 5 U/ml heparin, 100 U/ml of penicillin G, 100 μg/ml streptomycin, 0.25 μg/ml amphotericin B (Biological Industries, Cromwell, CT, U.S.A), and vascular endothelial cell growth supplement (ECGS) (Millipore, Billerica, MA, U.S.A.) in a humidified 37°C incubator. Other cells were maintained in DMEM (B16F10 cells) or RPMI1640 (GBM8901 cells) medium containing 10% FBS, 100 U/ml of penicillin G, 100 μg/ml streptomycin, and 0.25 μg/ml amphotericin B (Biological Industries, Cromwell, CT, U.S.A) in a humidified 37°C incubator. The murine LEC line SV-LEC was kindly provided by Dr. J.S. Alexander (Shreveport, LA). SV-LECs were cultured as previously described (34, 35)

### Lactate Dehydrogenase (LDH) Release Assay

The CytoTox96 non-radioactive cytotoxicity assay kit (Promega, Madison, WI, U.S.A.) was used to measure LDH leakage to quantify cytotoxicity as described previously (12).

### Cell Proliferation Assay

HUVECs (2 × 10^4^ cells per well) or SV-LECs (10^4^ cells per well) seeded in 96-well tissue culture plates were starved in M199 medium containing 2% FBS in the absence of endothelial cell growth supplements (HUVEC) or serum-free DMEM (SV-LEC) for 18 h. After starvation, cells were treated with MFB at indicated concentrations for 30 min, followed by the stimulation with VEGF-A (25 ng/ml) (HUVEC) or VEGF-C (100 ng/ml) (SV-LEC) for another 24 h. A BrdU Cell Proliferation kit (Millipore, Billerica, MA, U.S.A.) based on the colorimetric detection of the incorporation of BrdU was used to determine cell proliferation following the manufacturer’s instructions.

### Cell Migration (Scratch) Assay

HUVECs or SV-LECs were allowed to grow to confluence in 12-well tissue culture plates covered with (HUVEC) or without (SV-LEC) 0.1% gelatin (Sigma-Aldrich, St Louis, MO, U.S.A.). After starvation with M199 medium containing 2% FBS (HUVEC) or serum-free DMEM (SV-LEC) for 18 h, monolayer HUVECs and SV-LECs were wounded by scratching with pipette tips. Cells were washed with PBS, followed by the treatment with MFB at indicated concentrations with or without VEGF-A (25 ng/ml) or VEGF-C (100 ng/ml) for another 24 h. Cells were fixed with cold 4% paraformaldehyde and stained with 0.5% toluidine blue O. Microscope images were taken at 40× magnification by an *OLYMPUS* Biological Microscope digital camera (Yuan Li Instrument Co., Taipei, Taiwan). The gap closure rate was determined by comparing the sizes of the scratch area as a percentage of the values obtained with their respective controls at the beginning of experiments (time 0) using an Image J program (http://rsbweb.nih.gov/ij/index.html) (ImageJ).

### Transwell Invasion Assay

Transwell plate (Corning, NY, U.S.A.) was employed to perform the cell invasion assays. The bottom face of the insert membrane was coated with 0.2% gelatin. The bottom chambers were filled with M199 medium containing 2% FBS (HUVEC) or serum-free DMEM (SV-LEC) in the presence or absence of VEGF-A (25 ng/ml) (HUVEC) or VEGF-C (100 ng/ml) (SV-LEC). Cells (10^4^ cells per well) in 200 µL M199 medium containing 2% FBS (HUVEC) or serum-free DMEM medium (SV-LEC) with or without indicated concentrations of MFB were seeded in the top chambers. Cells were allowed to invade for 18 h. Non-invaded cells (on the top side of the insert membrane) were scraped with a cotton swab, and invaded cells were fixed with 4% paraformaldehyde and stained with 0.5% toluidine blue O. The cells were photographed under an inverted contrast phase light microscope (×40, Nikon, Japan). Stained HUVECs or SV-LECs that invaded through the insert membrane were quantified by counting in three random fields.

### Tube Formation Assay

The tube formation assay was performed as described previously (12). Matrigel basement membrane matrix (Becton Dickinson, Mountain View, CA, USA), was polymerized at 37°C for 30 min. HUVECs suspended in M199 medium containing 2% FBS with or without VEGF-A (25 ng/ml) or SV-LECs suspended in serum-free DMEM medium with or without VEGF-C (100 ng/ml) were seeded onto the Matrigel. After seeding, cells were treated with vehicle or indicated concentrations of MFB for 18 h (HUVEC) or 3 h (SV-LEC). Cells were photographed under an inverted contrast phase light microscope (×40, Nikon, Japan). The formed tube network was quantified by counting the number of tube sprout arch in three random fields.

### Animals

All animal care and experimental procedures complied with the recommendations in the Guide for the Care and Use of Laboratory Animals of the National Institutes of Health (NIH publication No. 85‐23, revised 1996) and were approved by the Taipei Medical University Laboratory Animal Care and Use Committee (Permit Number: LAC‐2018‐0432). Animal studies are reported in compliance with the ARRIVE guidelines (36).

### Aortic Ring Sprouting Assay

Six 8- to 10-week-old male Sprague-Dawley rats were purchased from National Laboratory Animal Center (Taipei, Taiwan) and used for the aortic ring sprouting assay. Rats were sacrificed using CO_2_ asphyxiation to dissect the aortic arches. The surrounding fibro-adipose tissues were removed. The aortas were thoroughly rinsed with M199 medium and cut into approximately 1 mm ring segments. In each experiment, the aortic rings obtained from one rat were utilized for different treatment groups. The aortic rings were immersed in Matrigel in the wells of a 48-well tissue culture plate. VEGF-A (25 ng/ml) with or without MFB was added to the wells. The aortic rings were cultured in a humidified 37°C incubator and the cultured medium was changed every 3 days. Growing sprouts of endothelial cells were photographed under an inverted contrast phase light microscope (×40, Nikon, Japan) on day 7. The sprouting area was determined on the computer-digitized images with Image-Pro Plus software (Media Cybernetics, Inc., Rockville, MD, USA) (Image-Pro Plus). An observer who was unaware of the treatment group assessed the sprouting area.

### *In Vivo* Matrigel Plug Angiogenesis Assay

The *in vivo* matrigel plug angiogenesis assay with nude_nu/nu_ mice as described previously (12) was used to determine MFB’s *in vivo* anti-angiogenic effects. 3- to 5-week old male nude_nu/nu_ mice with a body weight of about 20 g were obtained from National Laboratory Animal Center (Taipei, Taiwan) and used for the experiment presented in **Figure 3**. All the mice were housed (3 mice per cage) in clean specific pathogen-free (SPF) rooms (standard 12-hour dark/12-hour light cycle at 22°C) in Laboratory Animal Center of Taipei Medical University, and maintained on standard chow and autoclaved water. The cage floor was covered with *Bed* O’Cobs animal bedding (The Andersons, Maumee, OH, USA). All mice were randomly allocated to an individually ventilated cage (IVC) by vivarium staff, upon transfer from National Laboratory Animal Center (Taipei, Taiwan) into the animal housing room. All mice purchased from National Laboratory Animal Center were acclimatized in the animal housing room for 7 days before starting experiments. Mice were anesthetized with intraperitoneal pentobarbital (50 mg/kg). Once anesthesia was induced, an aliquot (500 μl) of Matrigel containing VEGF-A (100 ng/ml) with heparin (20 U) was injected subcutaneously into the right flank of each mouse (VEGF-A-induced angiogenesis model). In the other set of experiments, GBM8901 human glioblastoma cells were harvested and re-suspended in PBS. Cells (5×10^6^ cells) in a volume of 150 μl in the presence of heparin (20 U) were mixed with Matrigel (150 μl) and injected subcutaneously into the right flank of each mouse (Tumor cells-induced angiogenesis model). After implantation, animals were randomized to either the vehicle-treated control group or the treatment group, which received indicated concentrations of MFB. The treatment was administrated intraperitoneally once daily for 7 (VEGF-A-induced angiogenesis model) or 10 (GBM8901 cells-induced angiogenesis model) days. At the end of treatment, animals were sacrificed using CO_2_ asphyxiation, Matrigel plugs were removed and the surrounding tissues were trimmed. Isolated matrigel plugs were sonicated in PBS, allowing blood components to be dissolved in the solution. A Drabkin’s reagent kit (Sigma-Aldrich) was then used to determine the hemoglobin levels of derived supernatant according to the manufacturer’s instructions. The concentration of hemoglobin was calculated based on a set of hemoglobin standards.

### Melanoma Lung Metastatic Mouse Model

To determine the anti-metastatic effects of MFB, the melanoma lung metastatic model with C57BL/6 mice as described previously (12) was used. 6- to 8-week old male C57BL/6 mice with a bodyweight of about 35 g were obtained from National Laboratory Animal Center (Taipei, Taiwan) and used for the experiment presented in **Figure 3**. All the mice were housed (5 mice per conventional cage) in clean conventional animal housing rooms (standard 12-hour light/12-hour dark cycle at 22°C) in the Laboratory Animal Center of Taipei Medical University and maintained on standard chow and autoclaved water. The cage floor was covered with *Bed* O’Cobs animal bedding (The Andersons, Maumee, OH, USA). All mice were randomly allocated to a conventional cage by vivarium staff, upon transfer from National Laboratory Animal Center into the animal housing room. Mice were anesthetized with intraperitoneal pentobarbital (50 mg/kg). Once anesthesia was induced, mice were inoculated at the tail vein with B16F10 melanoma cells (10^6^ cells per mouse) suspended in 150 μl of sterile saline. Mice were randomized to either the vehicle-treated control group or the treatment group, which received MFB (10 mg/kg/day). The treatment was administrated intraperitoneally once daily for 18 days. At the end of treatment, mice were killed by CO_2_ asphyxiation and dissected. The lungs were collected and fixed in 10% paraformaldehyde. The number of the metastatic melanoma nodules was counted. Paraffin wax-embedded sections (5 μm) of lung tissue were stained with hematoxylin and eosin (H&E) staining and photographed under a microscope to assess metastatic nodules area. The metastatic nodules area was determined on the computer-digitized images with Image-Pro Plus software (Media Cybernetics, Inc., Rockville, MD, USA).

### Immunoblotting

Cells were harvested in a lysis buffer containing 10 mM Tris (pH 7.0), 140 mM NaCl, 0.5% NP-40, 0.05 mM pepstatin A, 0.2 mM leupeptin and 2 mM PMSF. Equal amounts of protein samples were subjected to SDS-PAGE and transferred onto an NC membrane (Pall Corporation, Washington, NY, U.S.A.). After blocking in a 5% non-fat milk-containing blocking buffer for 1 h, proteins were recognized using specific primary antibodies for 2 h, followed by horseradish peroxidase-conjugated secondary antibodies for another 1 h. To detect immunoreactivity, the enhanced chemiluminescence detection kit (Millipore, Billerica, MA, U.S.A.) was employed as per the manufacturer’s instructions. Quantitative data was obtained using a computing densitometer with a scientific imaging system (Biospectrum AC System, UVP).

### Molecular Docking Simulation

For docking simulation, the X‐ray crystallography structure for VEGF‐ A (PDB ID: 3V2A) (37) and VEGFR‐2 (PDB ID: 5EW3) (38) was obtained from RCSB Protein Data Bank. The preparation of protein was performed by Prepare Protein module in Discovery Studio 2.5 (DS2.5) to remove crystal water in crystallography structure, insert missing atoms in incomplete residues, protonate the structure of both proteins with Chemistry at Harvard Macromolecular Mechanics (CHARMM) force field (39), and optimize side‐chain conformation for residues with inserted atoms. For VEGF‐A, there are two small receptor cavities between VEGF‐A and VEGFR‐2. We combine these two small receptor cavities to define the binding site with the volume of 341.375 Å for VEGF‐A (12). The binding site of VEGFR‐2 was defined as the volume of the co‐crystallized compound in the X‐ray crystallography (12). Ligand Fit module in DS2.5 was performed to obtain the docking poses of the compound using a shape filter and Monte‐Carlo ligand conformation generation and optionally minimized with a CHARMM force field (39).

### Data and Statistical Analysis

To provide randomization and blinding in our experiments, in each experiment, the same cell was used to evaluate the effects of MFB versus the related control. Formal randomization was therefore not employed. Mice used in this study were randomly allocated to cages by vivarium staff and randomized into MFB‐ or vehicle‐treated groups before the treatment. The exact group size (n) was the same for each experiment in this study. Results are expressed as mean ± SEM; n ≥ 5, where “n” refers to independent values, and not replicates. To control for unwanted sources of variation and to reveal relevant trends, normalization was performed to compare the differences after the treatment. For MTT or BrdU assay, the viability or BrdU incorporation was expressed as fold changes over that of the vehicle‐treated cells, whose expression was set to 1 (100%). For the LDH assay, LDH release from lysis buffer‐treated cells (TL group) was considered to be 100% and LDH release from the VEGF‐A‐treated cells in the presence or absence of MFB was expressed as a percentage of the control. For immunoblotting, the levels of protein modification e.g., VEGFR2 or Akt phosphorylation) were normalized to that of unmodified protein (e.g., VEGFR2 or Akt). The status of protein modification was expressed as fold changes over that of the vehicle‐treated cells, whose expression was set to 1 (100%). The SEM has normalized appropriately. The status of protein modification was expressed by normalization that generates control values with no variance (SEM = 0) to reduce the effect of variation from different exposure of blotting, and such data are subjected to non‐parametric statistical analysis. The group data subjected to statistical analysis have a minimum of n = 5 independent samples per group in this study. Statistical analysis was performed using SigmaPlot 14 (Build 10.0.0.54; Systat Software, San Jose, CA, USA; SigmaPlot). Statistical comparisons between two groups were evaluated by the unpaired Student’s t‐test for parametric analysis or Mann-Whitney test for non‐parametric analysis. Statistical comparisons among more than two groups were evaluated by one‐way ANOVA with Tukey’s *post hoc* test for parametric analysis or Kruskal-Wallis test followed by Dunn’s multiple comparisons for non‐parametric analysis. *Post hoc* tests were run only if F achieved P <.05 and there was no significant inhomogeneity. A *P* value smaller than 0.05 was defined as statistically significant.

## Results

### MFB, a Novel 2-Aminobenzimidazole Derivative Suppressed Cell Proliferation, Migration, and Invasion in VEGF-A-Stimulated HUVECs

**Figure 1:**
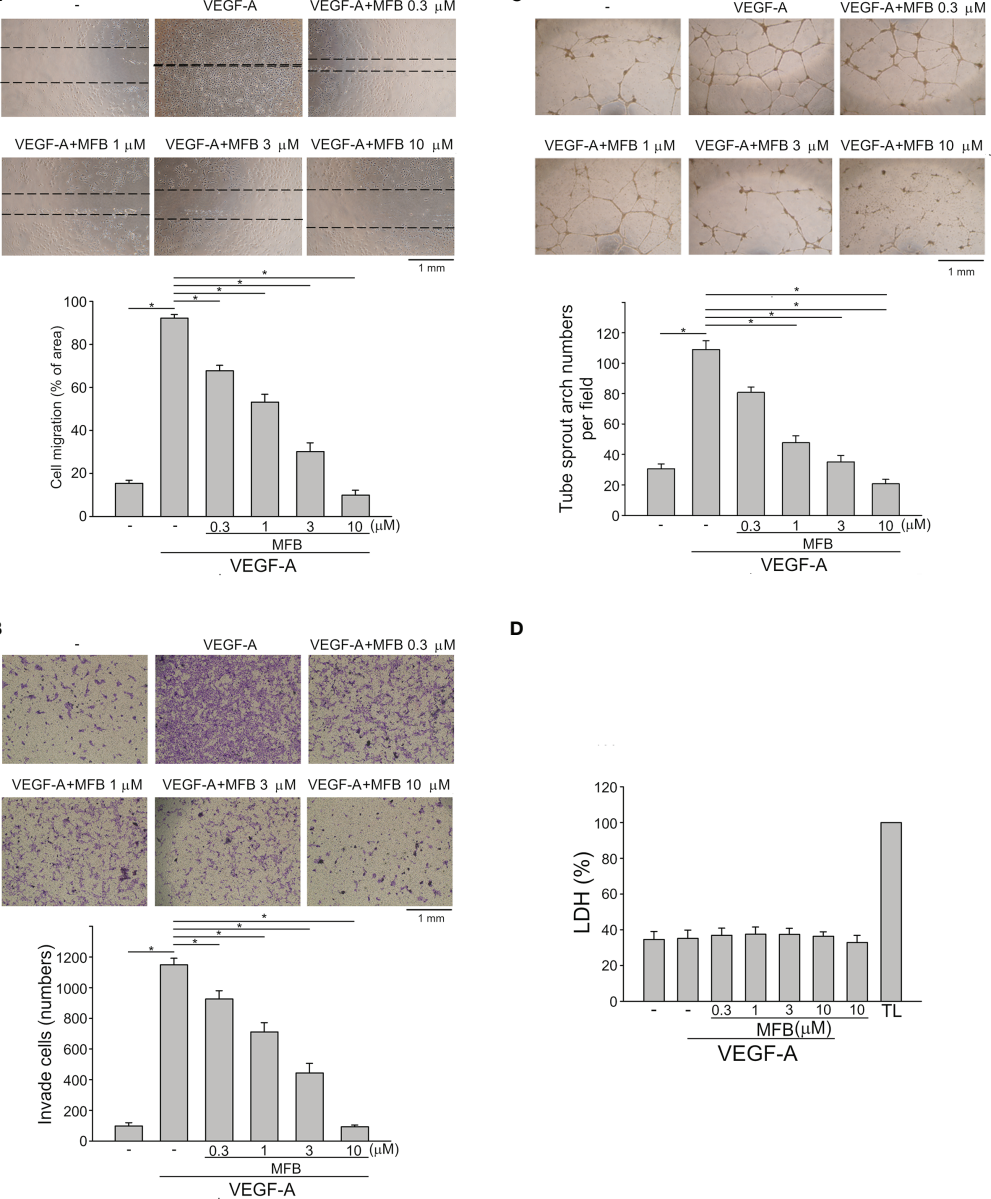
MFB suppressed VEGF-A-induced cell migration, invasion, and tube formation of HUVECs *in vitro* and reduced VEGF-A-induced aorta ring sprouting *ex vivo.* ***(*A*)*** HUVECs were starved in 2% FBS containing medium without ECGS for 18 h After starvation, cells were scratched and treated with vehicle or MFB in the absence or presence of VEGF-A for another 24 h. The rate of cell migration was determined. Each column represents the mean ± S.E.M. of six independent experiments * P <.05, significantly different from VEGF‐A alone; one‐way ANOVA, with Tukey’s post‐hoc test. **(B)** After starvation as described in **(A)**, a total of 2 x 10^4^ HUVECs were seeded in the top gelatin-coated chamber and treated with vehicle or MFB using VEGF-A as a chemo-attractant. After 18 h, the HUVECs that invaded through the gelatin-coated membrane barrier were stained and quantified. Each column represents the mean ± S.E.M. of eight independent experiments. *P <.05, significantly different from VEGF‐A alone; one‐way ANOVA, with Tukey’s post‐hoc test. **(C)** HUVECs were seeded on Matrigel in the presence of VEGF-A (200 ng/ml) with or without MFB. Cells were photographed under phase-contrast after 18 h Bar graphs show compiled data of average sprout arch numbers (n = 6). *P <.05, significantly different from VEGF‐A alone; one‐way ANOVA, with Tukey’s post‐hoc test. **(D)** Cells were stimulated with VEGF-A (25 ng/ml) with or without MFB for 18 h An LDH assay was used to determine the cytotoxicity of MFB. Cells were also treated with cell lysis buffer (total lysis, TL) to serve as a positive control (100%). Each column represents the mean ± S.E.M. of six independent experiments performed in duplicate. Technical replicates were used to ensure the reliability of single values for each experiment. *P <.05, significantly different from VEGF‐A alone; Kruskal–Wallis test.

The basic steps of angiogenesis involve cell proliferation, migration, invasion, and tube formation of vascular endothelial cells (15). To determine whether MFBre and MFB (**Figure S1A**), two novel 2-aminobenzimidazoles, exhibit anti-angiogenic properties, we examined their effects on VEGF-A-induced HUVEC proliferation using MTT and BrdU incorporation assays. After 18 h synchronization with starvation medium (M199 medium containing 2% FBS), HUVECs were stimulated by VEGF-A (25 ng/ml) in the absence or presence of these compounds for another 24 h. MFBre and MFB, like sunitinib or sorafenib (two multi-targeted RTK inhibitors), concentration-dependently decreased cell viability in VEGF-A-stimulated HUVECs as determined by MTT assay (**Figure S1B**). Results derived from BrdU incorporation assay also showed that both MFBre and MFB significantly inhibited VEGF-A-induced HUVEC proliferation (**Figure S1C**). Because MFB exhibited more prominent inhibitory effects, with an IC50 of approximately 0.55 μM, we sought to investigate the mechanisms underlying MFB’s anti-angiogenic actions in the following experiments. A would-healing migration assay was employed to determine whether MFB affects HUVEC motility after VEGF-A exposure. As shown in **Figure 1A**, MFB markedly inhibited VEGF-A-induced cell migration. Results derived from the transwell invasion assay demonstrated that MFB is also capable of suppressing VEGF-A-induced cell invasion (**Figure 1B**). We next explored whether MFB affects the tubular formation of HUVECs. HUVECs seeded on matrigel with or without MFB (1-10 μM) were stimulated by VEGF-A (25 ng/ml). As shown in **Figure 1C**, HUVECs exposed to VEGF-A for 24 h became elongated and formed a capillary-like tubular structure. These capillary-like tubes connected and created a mesh-like structure on the matrigel. However, MFB treatment resulted in impairment of capillary-like network formation in response to VEGF-A (**Figure 1C**). In addition, MFB at 1 to 10 μM was without effects on lactate dehydrogenase (LDH) release in HUVECs after 24 h exposure to VEGF-A (**Figure 1D**). These results indicate that MFB may impair VEGF-A-induced angiogenesis by suppressing proliferation, migration, invasion, and tube formation of vascular endothelial cells. It also suggests that MFB’s anti-angiogenic actions are not attributed to its cytotoxic effects on HUVECs.

### MFB Inhibited VEGF-C-Induced Cell Proliferation, Invasion, and Tube Formation of LECs

**Figure 2:**
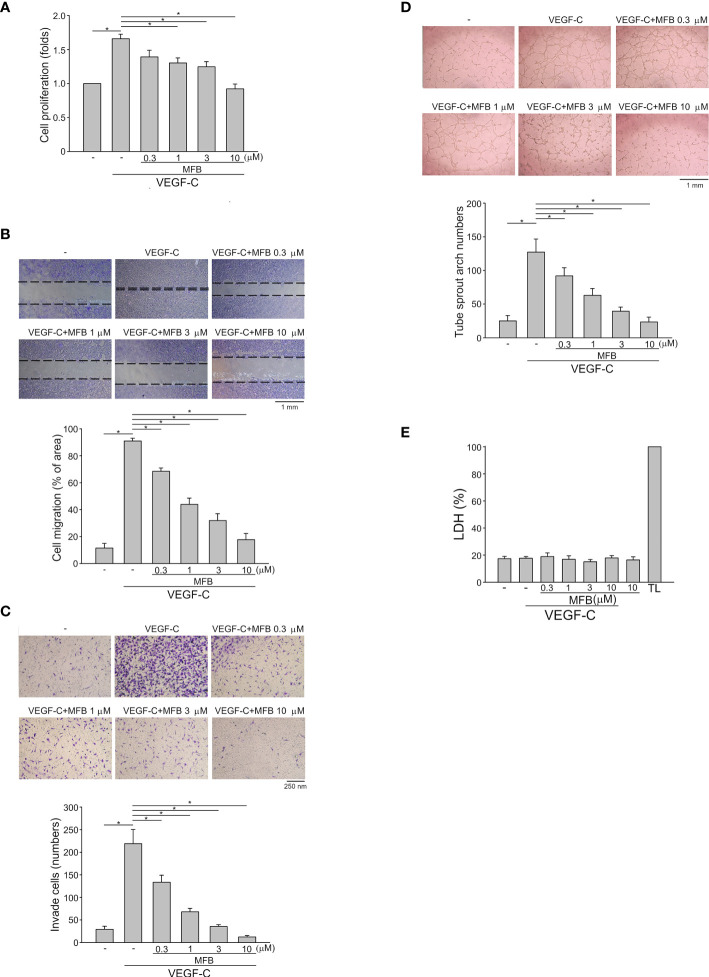
MFB suppressed VEGF-C-induced cell migration, invasion, and tube formation of SV-LECs **(A)** SV-LECs were starved in serum-free DMEM for 18 h. After starvation, cells were treated with MFB, followed by the stimulation with VEGF-C (100 ng/ml) for another 24 h. Cell proliferation was determined by a BrdU-based cell proliferation assay. Each column represents the mean ± S.E.M. of six independent experiments performed in duplicate. Technical replicates were used to ensure the reliability of singe values for each experiment. *P <.05, significantly different from VEGF‐A alone; Kruskal–Wallis test. **(B)** After starvation as described in **(A)**, cells were scratched and treated with vehicle or MFB in the absence or presence of VEGF-C for another 24 h. The rate of cell migration was determined. Each column represents the mean ± S.E.M. of six independent experiments * P <.05, significantly different from VEGF‐A alone; one‐way ANOVA, with Tukey’s post‐hoc test. **(C)** After starvation as described in **(A)**, a total of 2 x 10^4^ SV-LECs were seeded in the top gelatin-coated chamber and treated with vehicle or MFB using VEGF-C as a chemo-attractant. After 18 h, the SV-LECs that invaded through the gelatin-coated membrane barrier were stained and quantified. Each column represents the mean ± S.E.M. of ten independent experiments *P <.05, significantly different from VEGF‐A alone; one‐way ANOVA, with Tukey’s post‐hoc test. **(D)** SV-LECs were seeded on Matrigel in the presence of VEGF-C (100 ng/ml) with or without MFB. Cells were photographed under phase-contrast after 3 h. Bar graphs show compiled data of average sprout arch numbers (n = 6). *P <.05, significantly different from VEGF‐A alone; one‐way ANOVA, with Tukey’s post‐hoc test. **(E)** SV-LECs were stimulated with VEGF-C (100 ng/ml) with or without MFB for 18 h. An LDH assay was used to determine the cytotoxicity of MFB. Cells were also treated with cell lysis buffer (total lysis, TL) to serve as a positive control (100%). Each column represents the mean ± S.E.M. of six independent experiments performed in duplicate. Technical replicates were used to ensure the reliability of single values for each experiment. *P <.05, significantly different from VEGF‐A alone; Kruskal–Wallis test.

To determine whether MFB also exhibits anti-lymphangiogenic activity, an immortalized murine LEC line (SV-LEC), which retains their ‘lymphatic’ endothelial characteristics after repeated passages (35, 40), was employed. After 18 h synchronization with starvation medium (serum-free DMEM), SV-LECs were stimulated by VEGF-C (100 ng/ml) with or without MFB for another 24 h. MFB, like sunitinib or sorafenib, significantly reduced cell viability in SV-LECs exposed to VEGF-C as determined by MTT assay (**Figure S1E**). In addition, the percentage of BrdU-labeled SV-LECs significantly increased after a 24 h VEGF-C treatment. However, MFB reduced this increase in a concentration-dependent manner (**Figure 2A**). MFB significantly inhibited VEGF-C-induced cell migration as determined by a wound-healing assay (**Figure 2B**). MFB also reduced the number of invading cells penetrating the gelatin-coated filter barrier using VEGF-C as the chemoattractant (**Figure 2C**). Whether MFB affects the tubular formation of SV-LECs, another key step of lymphangiogenesis, was also determined. As shown in **Figure 2D**, cells became elongated and formed capillary-like structures after 3 h exposure to VEGF-C. MFB, however, significantly reduced the VEGF-C-induced capillary-like network formation of LECs on matrigel (**Figure 2D**). Moreover, MFB did not alter LDH release in the presence or absence of VEGF-C in SV-LECs (**Figure 2E**). These observations indicate that MFB may exhibit anti-lymphangiogenic properties through suppressing VEGF-C-induced cell proliferation, migration, invasion, and tubular formation of LECs.

### MFB Suppressed VEGF-A-Induced Microvessel Sprouting *Ex Vivo* and Inhibited Angiogenesis in *In Vivo* Models

**Figure 3:**
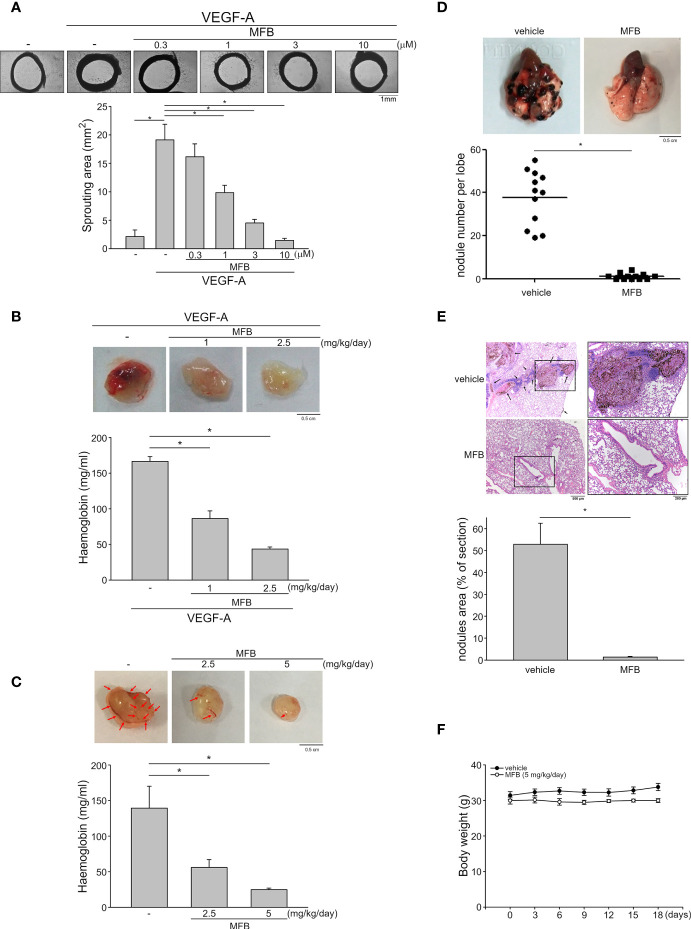
MFB reduced VEGF-A- or tumor cells-induced angiogenesis *in vivo* and suppressed murine melanoma B16F10 cell lung metastasis. ***(*A*)*** Rat aortic rings were placed in Matrigel with VEGF-A (25 ng/ml) in the presence or absence of MFB. The formation of microvessels sprout from aorta ring samples was determined on day 8. Bar graphs show compiled data of the average microvessels area (n=6). *P <.05, significantly different from VEGF‐A alone; one‐way ANOVA, with Tukey’s post‐hoc test. **(B)** Matrigel mixed with VEGF-A (100 ng/ml) was subcutaneously injected into the right flank of nude mice. After implantation, mice were administrated intraperitoneally with vehicle or MFB for 7 days. Matrigel plugs removed from the mice treated with vehicle or MFB were shown in the upper of the chart. Hemoglobin levels in the matrigel plugs were quantified. Each column represents the mean ± S.E.M. of eight plugs in each group. *P <.05, significantly different from VEGF‐A-treated group; one‐way ANOVA, with Tukey’s post‐hoc test. **(C)** Matrigel mix with GBM8901 cells was subcutaneously injected into the right flank of nude mice. After implantation, mice were administrated intraperitoneally with vehicle or MFB for 10 days. Matrigel plugs removed from the mice treated with vehicle or MFB were shown in the upper of the chart. Hemoglobin levels in the matrigel plug were quantified. Arrows point to newly formed microvessels in the matrigel plug. Each column represents the mean ± S.E.M. of six plugs in each group *P <.05, significantly different from the vehicle-treated group; one‐way ANOVA, with Tukey’s post‐hoc test. **(D)** B16F10 melanoma cells were injected into the tail vein of the C57BL/6 mice. These mice were intraperitoneally administrated with vehicle or MFB for 18 days. A representative photograph of lung metastatic foci after intravenous injection of B16F10 cells for 18 days was shown in the upper of the chart. Each symbol represents the average number of metastasis nodules in a lung lobe from an individual mouse (n=6 for each group). *P <.05, significantly different from the vehicle‐treated control group; student’s t‐test. **(E)** Representative lung hematoxylin and eosin (H&E) stained sections of metastases from vehicle- and MFB-treated mice. Arrows point to metastatic foci. Bar graphs show compiled data of the average area of metastasis nodules per section (n=6 for each group). *P <.05, significantly different from the vehicle‐treated control group; student’s t‐test. **(F)** The body weights of the mice were examined every 3 days within 18 days of treatment of vehicle or MFB. Values represent the mean ± S.E.M. (n = 6 for each group).

An *ex vivo* rat aortic ring sprouting assay was used to determine the anti-angiogenic effects of MFB. As shown in **Figure 3A**, VEGF-A markedly increased the sprouting microvessels to form a complex network around the aortic rings. This effect, however, was significantly reduced in the presence of MFB (1-10 μM) (**Figure 3A**). A matrigel plug angiogenesis assay was used to examine whether MFB is effective in suppressing VEGF-A- or tumor cells-induced angiogenesis *in vivo*. As shown in **Figure 3B**, the newly formed microvessels in the subcutaneously implanted matrigel plugs with VEGF-A (100 ng/ml) were markedly increased within 7 days. Intraperitoneal administration of MFB (1 or 2.5 mg/kg/day) caused a significant reduction in neovascularization as indicated by the pale color of the plugs removed from the MFB-treated mice when compared with those from vehicle-treated mice (**Figure 3B**, upper panel). This angiogenic response was also quantified by measuring the hemoglobin content of the plugs. As shown in **Figure 3B**, MFB treatment led to a significant reduction of VEGF-A-induced angiogenesis (**Figure 3B**, bottom panel). The *in vivo* inhibitory effects of MFB on tumor angiogenesis were also determined using a tumor cells-induced angiogenesis model. GBM8901 glioma cells mixed with matrigel were implanted subcutaneously into the flanks of mice. After implantation for 10 days with or without intraperitoneal administration of MFB (2.5 or 5 mg/kg/day), subcutaneously implanted matrigel plugs were harvested. As shown in **Figure 3C**, GBM8901 cells increased neovascularization in the matrigel plugs and this effect, however, is reduced by MFB (**Figure 3C**, upper panel). The hemoglobin content of the plugs was determined to quantify angiogenesis. As shown in **Figure 3C**, MFB significantly reduced tumor cells-elicited angiogenesis *in vivo* as compared with the vehicle-treated control group (**Figure 3C**, bottom panel). We also performed the immunohistological analysis to examine the lymphatic vessel and microvessel density *via* anti-LYVE-1 and anti-CD31 staining respectively. As shown in **Figure S2**, MFB suppressed tumor cells-induced lymphatic vessel growth significantly and was also indicated with a remarkable decline in microvessel area as compared with vehicle-treated group. These results indicate that the systemic administration of MFB is capable of suppressing tumor cells-induced angiogenesis and lymphangiogenesis *in vivo*.

### MFB Inhibited Lung Metastasis of B16F10 Melanoma Cells

A pulmonary metastatic model of murine melanoma was used to examine the inhibitory effects of MFB on tumor metastasis *in vivo*. B16F10 murine melanoma cells were inoculated into the tail vein of C57BL/6 mice to generate multiple lung metastases. Lungs of mice with or without intraperitoneal administration of MFB (5 mg/kg/day) were removed after inoculation for 18 days. As shown in **Figure 3D**, there are multiple visible melanoma nodules with different sizes on the lung surface of the vehicle-treated mice (**Figure 3D**, upper panel). However, the lungs of the MFB-treated mice exhibited markedly smaller and fewer metastatic nodules (**Figure 3D**, upper panel). The number of metastatic melanoma nodules was counted to quantify the degree of lung metastases. As shown in **Figure 3D**, MFB significantly reduced the number of lung metastases as compared with the vehicle-treated control group (**Figure 3D**, bottom panel). Results derived from histological analysis of lung tissues with hematoxylin and eosin (H&E) staining also showed that MFB markedly reduced lung metastatic nodular areas (**Figure 3E**). Moreover, MFB treatment had no significant effects on mouse body weight as compared to the vehicle-treated control group (**Figure 3F**). These observations indicate that MFB exhibits anti-metastatic properties *in vivo*.

### MFB Suppressed VEGF-VEGFR Signaling in HUVECs and SV-LECs

**Figure 4:**
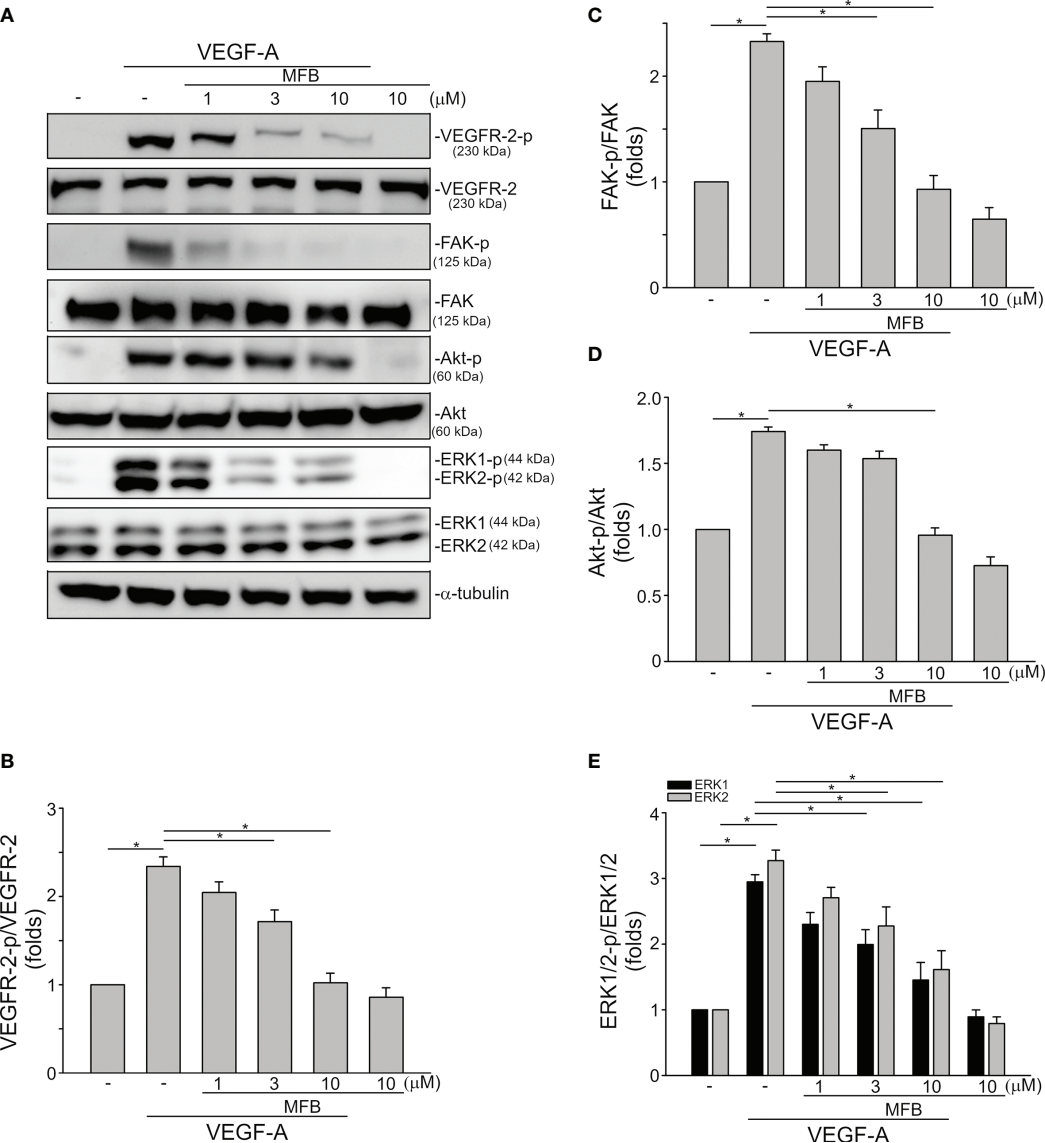
MFB suppressed VEGF-A/VEGFR-2 signaling in HUVECs. **(A)** HUVECs were treated with MFB for 30 min, followed by the stimulation with VEGF-A (25 ng/ml) for another 5 (VEGFR-2) or 30 (FAK, Akt, and ERK1/2) min. The phosphorylation status of VEGFR-2, FAK, Akt, and ERK1/2 was determined by immunoblotting. The compiled results of VEGFR-2 Tyr1175 **(B)**, FAK Tyr397 **(C)**, Akt Ser473 **(D)**, and ERK1/2 Thr202/Tyr204 **(E)** phosphorylation are shown. Each column represents the mean ± S.E.M. of six independent experiments. *P <.05, significantly different from VEGF‐A alone; ANOVA on Ranks, with Kruskal-Wallis test.

**Figure 5:**
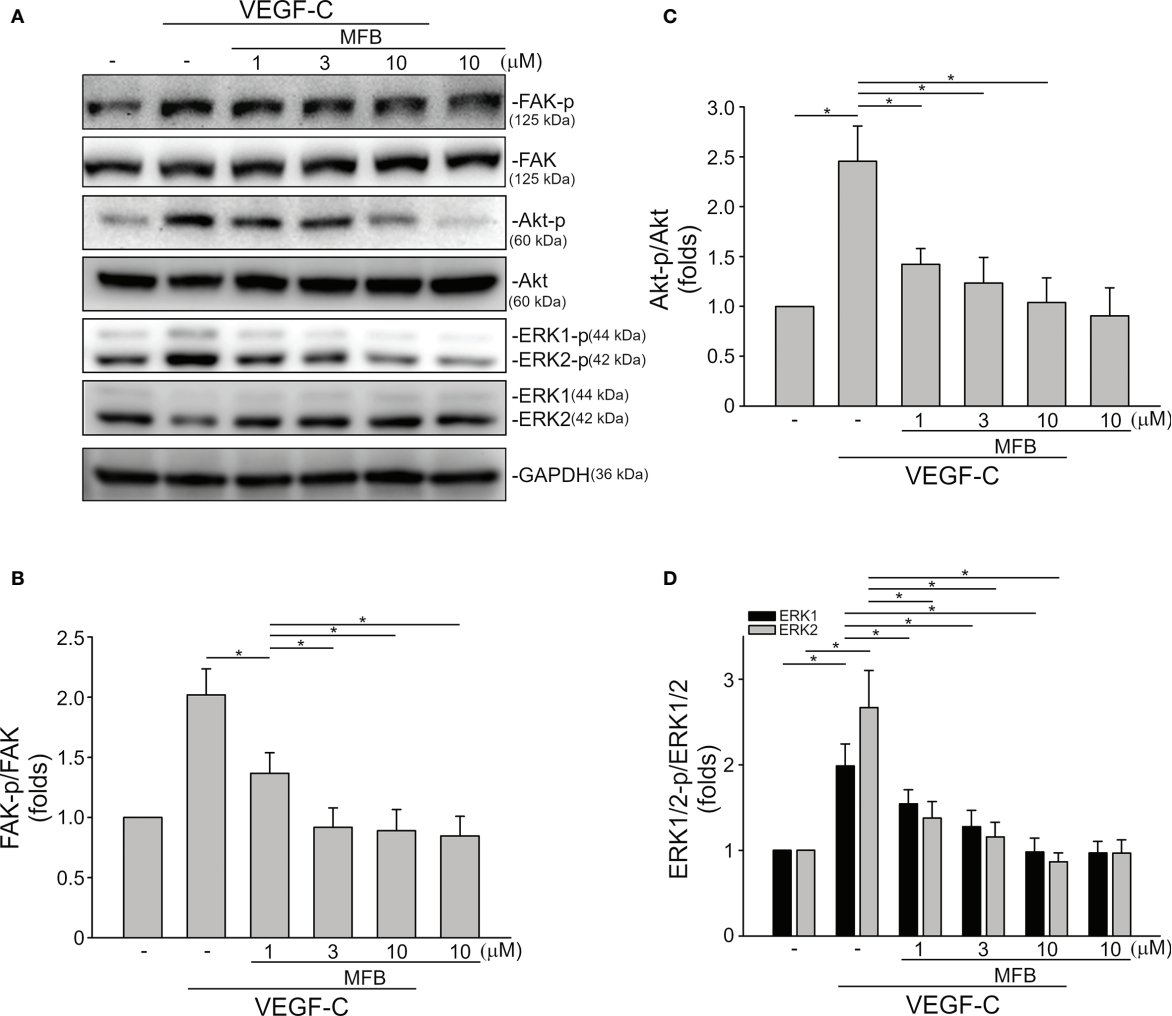
MFB reduced FAK, Akt, and ERK1/2 phosphorylation in VEGF-C-stimulated SV-LECs. **(A)** SV-LECs were treated with MFB for 30 min, followed by the stimulation with VEGF-C (100 ng/ml) for another 30 min. The effects of MFB on the phosphorylation of FAK, Akt, and ERK1/2 were determined by immunoblotting. The compiled results of FAK Tyr397 **(B)**, Akt Ser473 **(C)**, and ERK1/2 Thr202/Tyr204 **(D)** phosphorylation are shown. Each column represents the mean ± S.E.M. of six independent experiments. *P <.05, significantly different from VEGF‐C alone; ANOVA on Ranks, with Kruskal-Wallis test.

VEGF-A signaling through VEGFR-2 plays a prominent role in blood vessel homeostasis and vascular diseases (41). Activation of VEGFR-2 entails phosphorylation of its tyrosine residues, primarily Tyr 1175 (13). We thus examined whether MFB alters VEGFR-2 Tyr 1175 phosphorylation in HUVECs exposed to VEGF-A. As shown in **Figure 4**, MFB significantly inhibited VEGF-A-induced VEGFR-2 Tyr 1175 phosphorylation (**Figures 4A, B**). MFB also reduced the phosphorylation of FAK (**Figures 4A, C**), Akt (**Figures 4A, D**) and ERK1/2 (**Figures 4A, E**), the signaling pathways downstream of VEGFR-2, in VEGF-A-stimulated HUVECs. ERK1/2 and Akt signaling cascades have recently been recognized as part of major pathways involved in lymphangiogenesis (42, 43). We examined the effects of MFB on ERK1/2, Akt, as well as FAK phosphorylation in VEGF-C-stimulated SV-LECs. As shown in **Figure 5A**, VEGF-C significantly increased phosphorylation of FAK (**Figure 5B**), Akt (**Figure 5C**) and ERK1/2 (**Figure 5D**) in SV-LECs. This effect, however, was reduced in the presence of MFB, suggesting that MFB is also capable of affecting VEGF-C signaling in LECs. Together these observations reveal that targeting VEGF/VEGFR signaling is responsible for the anti-angiogenic and anti-lymphangiogenic activities of MFB.

### Computational Modeling of the Interactions Between MFB and VEGF-A or VEGFR-2

**Figure 6:**
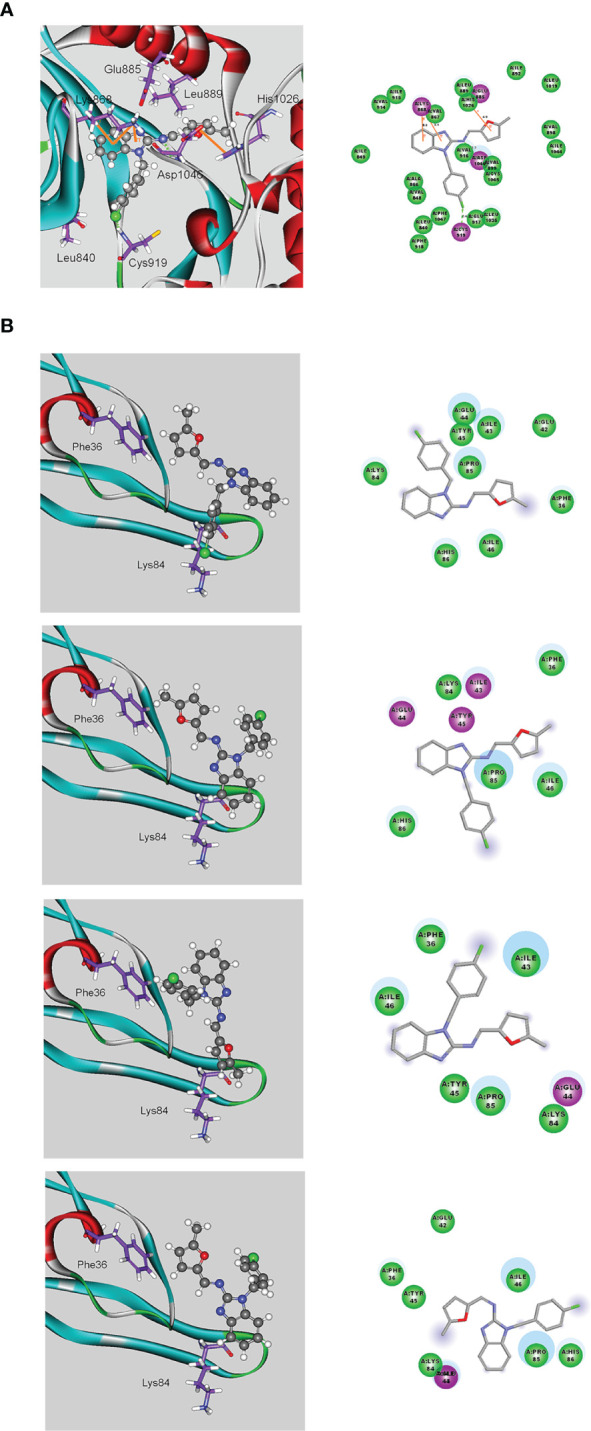
Molecular docking simulation analysis of MFB. **(A)** Molecular modeling of the interactions between MFB and VEGFR-2. The graph shows the docking pose of VEGFR-2 with MFB (left panel). The docking pose of VEGFR-2 with MFB using a 2D ligand-protein interaction diagram is shown in the right panel. Orange represents π-cation interactions **(B)** Molecular modeling of the interactions between MFB and VEGF-A. The graph shows the docking pose of VEGF-A with MFB (left panel). The docking pose of VEGF-A with MFB using 2D ligand-protein interaction diagrams is shown in the right panel.

We next examined the effects of MFB and sunitinib on the intrinsic tyrosine kinase activity of VEGFR-2. Similar to the previous report (12), we noted that sunitinib markedly reduced purified recombinant VEGFR-2 tyrosine kinase activity as determined by an *in vitro* kinase assay (**Figure S3**). To our surprisingly, MFB at a high concentration of 10 μM only slightly affected the tyrosine kinase activity of VEGFR-2 (**Figure S3**). It raises the possibility that MFB may interact with VEGF-A or VEGFR-2 to antagonize VEGF-A/VEGFR-2 signaling. Therefore, we analyzed the possible interactions between MFB and VEGF-A or VEGFR-2 using molecular docking simulations. The binding site of VEGFR-2 was defined as the volume of the co-crystallized compound, while the binding site of VEGF-A was defined by two receptor cavities between VEGFR-2 and VEGF-A (12). The docking score and scoring results are demonstrated in **Table 1**. As shown in **Figure  6A**, the docking pose of MFB has π-cation interactions with VEGFR-2 Lys868 and His1026 residues (**Figure 6A**, left panel). Similar to the docking pose of the co-crystallized compound, MFB forms a stable docking pose with VEGFR-2 in the X-ray crystallography. The 2D ligand-protein interaction diagram between VEGFR-2 and MFB was also illustrated (**Figure 6A**, right panel). In addition, there are four different possible docking poses of MFB for VEGF-A (**Figure 6B**). These poses lay in different positions of the binding site and have no hydrogen bonding or π-cation interactions with VEGF-A. It appears that MFB may not have a stable docking pose for VEGF-A. We also examined whether VEGF-A binding to VEGFR in HUVECs was altered in the presence of MFB. As shown in **Figure S4**, MFB reduced VEGF-A binding to VEGFR in HUVECs as determined by flow-cytometric analysis. Together these findings suggest that MFB may bind to VEGFR-2 rather than VEGF-A to interfere with VEGF-A/VEGFR-2 signaling.

**Table 1:** Scoring functions of MFB with VEGF-A and VEGFR-2.

Protein	Compound	Pose	-PLP1	-PLP2	-PMF
VEGFR-2	MFB	1	114.29	109.72	109.19
VEGF-A	MFB	1		40.1	23.64
	MFB	2	46.36	37.12	31.01
	MFB	3	49.19	40.17	18.4
	MFB	4	47.66	38.87	35.09

Triple consensus scoring: PLP1, PLP2, and PMF. Piecewise Linear Potential (PLP); Potential of Mean Force (PMF).

## Discussion

Cancer remains the leading cause of death with increasing incidence over the past few decades worldwide. Although the burden of cancer has increased, the number of cancer survivors has also increased in developed countries in response to the advances in treatment options. However, it remains crucial to develop novel therapeutic agents or strategies that more effectively improve outcomes in patients with cancer. The majority of cancer-related deaths are caused by metastasis. Therefore, the essential role of angiogenesis and lymphangiogenesis in tumor metastasis makes it a promising target for anti-tumor drug development (15, 44). Targeting tumor vasculature also improves the potency of chemotherapy (45) and enhances anti-cancer immunity (46). Currently, anti-angiogenic therapy has been used clinically in the adjuvant setting of cancer patients (47). Efforts to develop novel anti-angiogenic or anti-lymphangiogenic agents primarily focused on targeting VEGF/VEGFR signaling, a central regulator, despite the presence of other signaling pathways involved in angiogenesis and lymphangiogenesis (7, 20, 26, 27). Increasing evidence is showing that benzimidazoles readily interact with signaling molecules and exhibit a broad spectrum of biological activities including anti-cancer properties (48). In this study, we have identified a 2-aminobenzimidazole-containing small molecule, MFB, as a potent inhibitor of angiogenesis and lymphangiogenesis. We showed that MFB significantly reduced VEGF-A- or tumor cells-induced angiogenesis in *in vivo* models. We also noted that MFB markedly reduced the number and size of melanoma lung metastases in a mouse model of metastasis. Moreover, we demonstrated that MFB may target VEGF-A/VEGFR-2 and VEGF-C/VEGFR-3 signalings to exhibit their anti-angiogenic and lymphangiogenic properties.

VEGFR-2 is the major transducer of VEGF signals in angiogenesis. VEGF-A binding to VEGFR-2 causes its dimerization, followed by phosphorylation of specific tyrosine residues, particularly Tyr1175 (Y1175), and triggering angiogenic signaling (49). We noted that MFB suppresses VEGF-A-induced VEGFR-2 Y1175 phosphorylation and its downstream signaling events. MFB also exhibited anti-angiogenic activity *in vivo*. However, MFB only slightly reduced VEGFR-2 tyrosine kinase activity as determined by an *in vitro* kinase assay (**Figure S3**). Whether MFB exhibits inhibitory effects on other receptor tyrosine kinases (RTKs) remains to be investigated. Results derived from the molecular docking simulation analysis revealed that MFB may antagonize VEGF-A/VEGFR-2 signaling through binding to VEGFR-2 rather than VEGF-A. Together these findings suggest that MFB may not directly inhibit VEGFR-2, but may represent as a VEGFR-2 antagonist to interfere with VEGF-A/VEGFR-2 signaling and inhibit angiogenesis.

Besides suppressing angiogenic actions of VEGF-A, we demonstrated that MFB also inhibits VEGF-C-induced lymphangiogenesis. The binding of VEGF-C to VEGFR-3 leads to the activation of ERK and Akt signaling cascades, which are required for LEC survival, migration, and proliferation (50). In addition, FAK not only plays a regulatory role in angiogenesis but also contributes to VEGF-C-induced lymphangiogenesis *in vitro* and *in vivo* (51). In agreement with these observations, we noted that MFB reduces VEGF-C-induced FAK, ERK, and Akt phosphorylation in SV-LECs. MFB may likely target VEGF-C signaling to inhibit lymphangiogenesis. However, the precise mechanism underlying MFB’s anti-lymphangiogenic actions remains to be investigated. We noted that MFB at 10 μM only inhibits VEGFR-3 kinase activity by approximately 6% as determined by Z’-LYTE kinase assay (unpublished data). Due to the lack of a suitable docking pose to predict the docking score, whether MFB is capable of binding to and antagonizing VEGFR-3 remains unclear. Whereas, the docking pose of MFB has π-cation interactions with VEGF-C Trp126 residue as determined by a molecular docking simulation analysis (**Figure S5**). **Table S1** shows the docking score and scoring results (**Table S1**). In addition, VEGF-C is capable of binding to both VEGFR-2 and VEGFR-3 and regulating the formation of vascular and lymphatic vessels (50, 52). Together these observations raise the possibility that MFB may bind to VEGF-C, at least in part, to counterbalance VEGF-C/VEGFR-3 and VEGF-C/VEGFR-2 actions regarding lymphangiogenesis.

Benzimidazoles are a class of bioactive heterocyclic, aromatic compounds that are present in a variety of natural or synthetic medicinal compounds. Benzimidazole is thus recognized as a key pharmacophore in the field of drug discovery. In addition to other biological actions and beneficial effects, benzimidazoles also possess potential anti-proliferative and anti-tumor activities (53, 54). We noted that the 2-aminobenzimidazole-based compound, MFB, reduced cell viability in SW480 colorectal cancer, A549 lung cancer, B16F10 melanoma and GBM8901 glioma cells. However, the cell viability of non-tumor Hacat keratinocytes was not altered in the presence of MFB (**Figure S6**). MFB likely has additional properties with anti-tumor effects. Whether MFB is also effective in decreasing cell viability in other cancer types and the underlying mechanisms remain to be delineated. The mechanism of the difference in sensitivity of MFB between tumor cells and non-tumor Hacat keratinocytes remains to be investigated. It has been described that cell doubling time may affect sensitivity to chemotherapeutic agents (55). In addition, several studies have suggested that VEGFRs may be expressed by tumor cells, although VEGFRs are regarded as endothelial receptors. VEGF could participate in tumor growth and metastasis *via* autocrine and paracrine mechanisms (56–58). VEGFs and VEGFRs are highly expressed in melanoma cells. Elevated serum VEGF levels is correlated with progression of malignant melanoma and poor prognosis in patients with melanoma (59). Besides melanoma, VEGF signaling is also crucial for the survival and growth of non-small cell lung cancer (NSCLCs) (60) and glioblastoma multiforme (GBM) (61). Moreover, Hasan et al. (62) showed that inhibition of VEGF signaling using bevacizumab could induce cell senescence in colorectal cancer cells. Additional works are needed to characterize whether MFB’s actions in tumor cells involve VEGF/VEGFR signaling blockade. Whether MFB-reduced tumor cell viability is attributed to VEGFR-independent signaling cascades is also worth to be further investigated.

Small-molecule angiogenesis inhibitors are expected to have fewer and milder adverse effects than widely used conventional chemotherapeutics. Toxicities commonly attributed to anti-angiogenic agents include proteinuria, hypertension, impairment of wound-healing, hypersensitivity, and vascular complications such as hemorrhage and thromboembolism (63). Regarding the *in vivo* effects of MFB on hemostasis or the risk of hemorrhage, we noted that intraperitoneal administration with MFB for 10 days was without effects on tail-bleeding time, a commonly used model to analyze hemostasis. Sunitinib, however, prolonged the tail-bleeding time (**Figure S7**). MFB may likely be safer in posing a lower risk of bleeding. Moreover, MFB treatment significantly reduced VEGF-A- or tumor cells-induced angiogenesis but did not alter the bodyweight of the mice. These observations suggest that MFB may represent an effective anti-angiogenic agent with less toxicity.

In conclusion, we showed in this study that MFB, a novel 2-aminobenzimidazole-based compound, exhibits anti-angiogenic and anti-lymphangiogenic properties *via* targeting VEGF-A/VEGFR-2 signaling in vascular endothelial cells and VEGF-C/VEGFR3 signaling in LECs. MFB may also have additional properties with anti-tumor effects. The mechanisms underlying its anti-tumor actions remain to be further investigated, but together these findings support the potential of MFB as a valuable lead in developing anti-angiogenic, anti-lymphangiogenic, and/or anti-tumor agents for future oncologic therapy.

## Data Availability Statement

The original contributions presented in the study are included in the article/**Supplementary Material**. Further inquiries can be directed to the corresponding author.

## Ethics Statement

The animal study was reviewed and approved by Taipei Medical University Laboratory Animal Care and Use Committee.

## Author Contributions

M-JH: Conceptualization, Methodology, Formal analysis, Investigation, Writing - Original Draft; H-KC: Investigation, Methodology, Formal analysis, Resources; C-YC: Conceptualization, Resources; J-CL: Methodology, Resources, Investigation; J-YG: Methodology, Formal analysis; Y-HH: Conceptualization, Validation; JB-KH: Conceptualization; GL: Conceptualization; S-WH: Conceptualization, Methodology, Investigation, Validation, Writing - Review and Editing, Supervision. All authors contributed to the article and approved the submitted version.

## Funding

This work was supported by the Ministry of Science and Technology of Taiwan [MOST 109-2314-B-038-129, MOST 109-2320-B-038-045-MY3, and MOST 110-2320-B-038-035-MY3]; the Chi Mei Medical Center, Tainan, Taiwan [108CM-TMU-11].

## Conflict of Interest

The authors declare that the research was conducted in the absence of any commercial or financial relationships that could be construed as a potential conflict of interest.

## Publisher’s Note

All claims expressed in this article are solely those of the authors and do not necessarily represent those of their affiliated organizations, or those of the publisher, the editors and the reviewers. Any product that may be evaluated in this article, or claim that may be made by its manufacturer, is not guaranteed or endorsed by the publisher.

## Abbreviations

ERK, extracellular signal-regulated kinase; FAK, focal adhesion kinase; HUVEC, human umbilical vascular endothelial cell; LEC, lymphatic endothelial cell; RTK, receptor tyrosine kinase; VEGF, vascular endothelial growth factor.
