# Supplementary material for: Anti-Angiogenetic and Anti-Lymphangiogenic Effects of a Novel 2-Aminobenzimidazole Derivative, MFB
Source: Front Oncol. 2022 Jun 20;12:862326. doi: 10.3389/fonc.2022.862326 (PMC9251317; doi:10.3389/fonc.2022.862326)
Supplement: Supplementary file 1 [file DataSheet_1.doc]

**Supplement Information**

**Supplement Methods**

***Synthesis of MFB:***

***
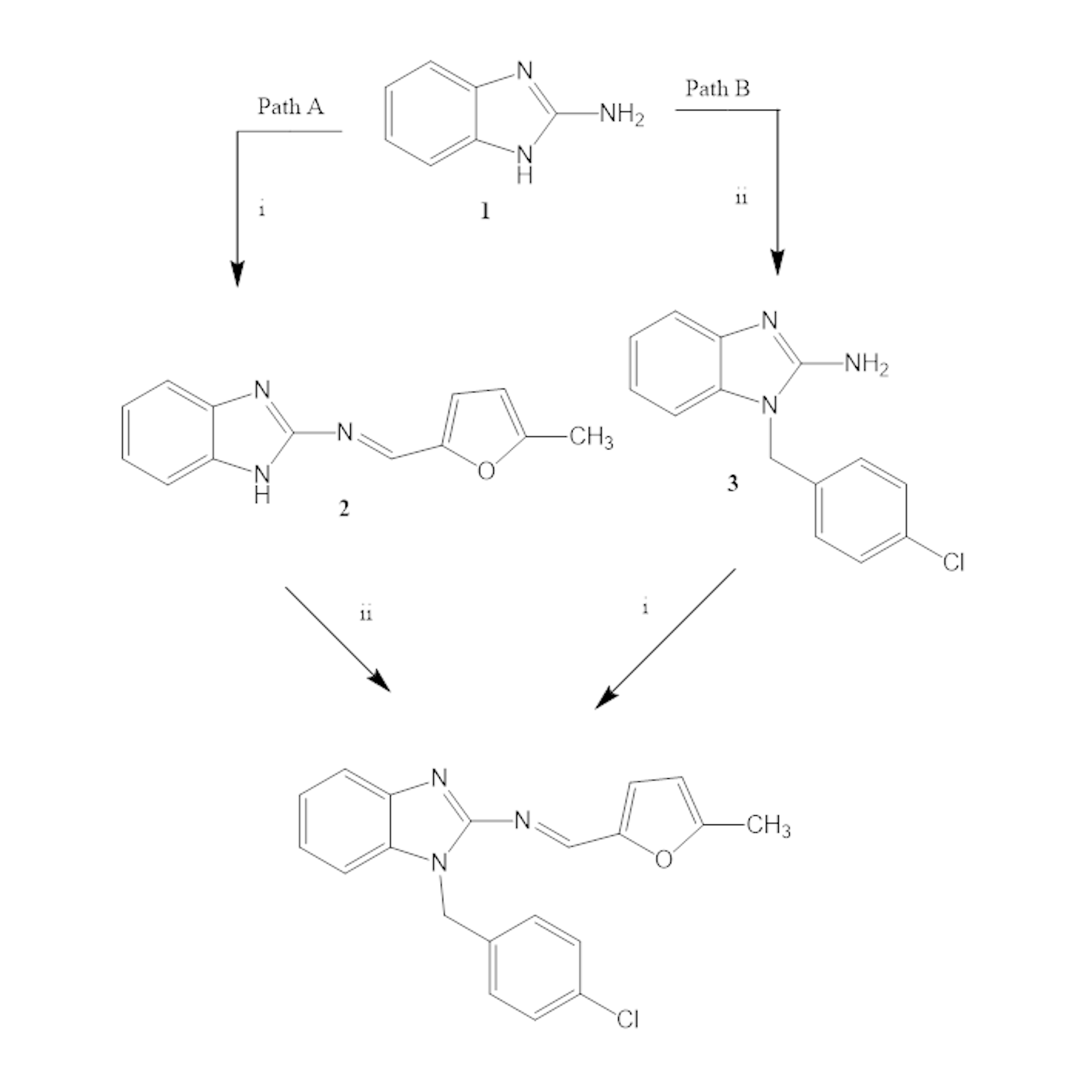
***

**Synthesis of *1-(4-chlorobenzyl)-2-(5-methyl-2-furfurylideneamino)-benzimidazole (MFB)***

There are two routes to prepare **MFB** from the starting material 2-aminobenzimidazole (compound **1**). In path A, 5-methyl-2-furaldehyde and compound **1** are dissolved in butanol and heat to reflux to give the product compound **2**. Then the compound **2** reacts with 4-chlorobenzyl chloride in the presence of KOH to give **MFB**. From path B, 1-(4-chlorobenzyl)-2-aminobenzimidazole (compound **3**) was obtained by reacting 2-aminobenzimidazole with 4-chlorobenzyl chloride. And then compound **3** was condensed with 5-methyl-2-furaldehyde affording **MFB**. Path B and path A have the same synthesis step but path B gives better yield of **MFB** than does path A.

**MFB** [1-(4-chlorobenzyl)-2-(5-methyl-2-furfurylideneamino)benzimidazole]: Yield: 20.2%; yellow needle crystals; mp: 132-133 °C; IR (KBr) ʋ (cm-1): 1570,1616 (C=C,C=N); 1H NMR (400 MHz, DMSO-d6)δ (ppm): 2.39 (3H, s, 5’-CH3), 5.52 (2H, s, -CH2), 6.44 (2H, d, J=3.2 Hz, H-4’), 7.09-7.17 (2H, m, H-5,6), 7.23 (2H, d, J=8.4 Hz, H-2”,6”), 7.33 (2H, d, J=8.4 Hz, H-3”,5”), 7.38-7.40 (2H, m, H-3’,7), 7.54 (1H, d, J=7.6 Hz, H-4), 9.13 (1H, s, N=CH); 13C NMR (100 MHz, DMSO-d6) δ (ppm): 13.50, 44.22, 110.05(d), 118.29, 121.71, 122.10, 124.46, 128.28, 128.67, 131.79, 134.10, 135.85, 140.97, 149.66, 151.11, 154.75, 158.79; MS (EI, 70 eV) m/z: 349 (M+); HRMS (EI) m/z: calc. for C20H16ClN3O: 349.0982; found: 349.0989. The purity of **MFB,** which is confirmed by HPLC spectrum, is greater than 95 %.

***Cell culture***

SW480 colorectal cancer, A549 lung cancer and B16F10 melanoma cell lines were obtained from the Bioresource Collection and Research Center (Hsinchu, Taiwan). The human immortalized keratinocyte cell line, Hacat was from DKFZ (Heidelberg, Germany). The cells were maintained in DMEM containing 10 % FBS, 100 U/ml of penicillin G, 100 μg/ml streptomycin, 0.25 μg/ml amphotericin B in a humidified 37 °C incubator.

***Immunohistochemical analysis***

Matrigel plugs isolated from the GBM8901 cells-induced angiogenesis model were fixed with 4 % paraformaldehyde (PFA). The matrigel plugs were dehydrated through series of ethanol solutions using Shandon Excelsior ES tissue processor (Thermo Fisher Scientific, Waltham, MA, U.S.A.). The dehydration and clearing steps were as follows: 15 min in 70 % ethanol, 15 min in 90 % ethanol, 15 min in 100 % ethanol, 15 min in 100 % ethanol, 20 min in 100 % ethanol, 30 min in 100 % ethanol and 18 h in xylene. The plugs were paraffin-embedded and sectioned for histological analysis. Immunohistochemical analysis was performed using a BOND-MAX Fully Automated IHC and ISH Staining System (Leica, USA). Sections were mounted on silanized slides and allowed to dry overnight at 37 °C. After deparaffinization and rehydratation, slides were incubated with 3 % hydrogen peroxide solution for 5 min. After a washing procedure with the supplied buffer, matrigel plug sections were repaired for 40 min with ethylenediamine tetraacetic acid. The slides were incubated with the anti-CD31 (1:4000, ab281583, Abcam, Cambridge, MA, USA) or anti-LYVE1 antibody (1:1000, ab33682, Abcam, Cambridge, MA, USA) for 60 min at 37 °C and then overnight at 4 °C. Antibody binding was visualized using stable diaminobenzidine after incubation with the peroxidase-conjugated goat anti-rabbit antibody (The Jackson Laboratory, Sacramento, CA, USA). Slides were counterstained with hematoxylin and microscope images were taken at 40× magnification by an OLYMPUS Biological Microscope digital camera (Yuan Li Instrument Co., Taipei, Taiwan). The blood vessel (CD31^+^) or lymphatic vessel (LYVE1^+^) area was determined on the computer-digitized images using an Image J program (<http://rsbweb.nih.gov/ij/index.html>) ([ImageJ](https://imagej.net/)).

***In vitro VEGFR-2 kinase assay***

A Kinase-Glo Plus luminescence kinase assay kit (Promega, Madison, WI, U.S.A.) was used to examine the effects of MFB on purified recombinant VEGFR-2 tyrosine kinase activity. The amount of ATP remaining in solution after a kinase reaction is quantitated to measure the kinase activity. The amount of kinase activity is inversely correlated with the amount of ATP present (the luminescent signal). Sunitinib and MFB were diluted to 100 µl in 10 % DMSO and 5 µl of the dilution was added to a 50 µl reaction to make a final concentration of 1% DMSO in all of reactions. All of the enzymatic reactions were performed at 30 ℃ for 30 minutes. The 50 µl reaction mixture contains 10 µM ATP, 0.1 mg/ml BSA, 10 mM MgCl_2_, 1 mM DTT, 40 mM Tris, pH 7.4, Kinase substrate and VEGFR-2. After the enzymatic reaction, 50 µl of Kinase-Glo Plus Luminescence kinase assay solution (Promega, Madison, WI, U.S.A.) was added to each reaction and incubated the plate at room temperature for 10 minutes. Luminescence signal was determined using a BioTek Synergy 2 microplate reader. The difference between luminescence intensities in the absence (Lut) and in the presence (Luc) of VEGFR-2 was defined as 100 % activity (Lut - Luc). Using luminescence signal (Lu) in the presence of the compound, % activity was calculated as: % activity = {(Lut - Lu)/(Lut - Luc)}×100%, where Lu= the luminescence intensity in the presence of the compound.

***Flow-cytometry***

Cells were treated with MFB at indicated concentrations for 24 h. Cells were washed twice with PBS and fixed in 70 % ethanol at 0 °C for another 24 h. After washing with phosphate-citric acid buffer, cells were stained by staining buffer (25 μg/ml PI, 100 μg/ml RNase A and 0.1 % Triton X-100) in the dark for 30 min. Flow-cytometry was performed using the Attune flow cytometer (Attune NxT, Invitrogen, Carlsbad, CA, U.S.A.). The percentage of PI-stained cells in the subG1 (Apoptosis, Apo), G0/G1, S or G2/M region was analyzed using the ModFit (BD Biosciences, San Jose, CA, U.S.A.) program.

***VEGF-A binding analysis***

A human VEGF-A biotinylated fluorokine kit (R&D Systems) was employed to perform VEGF-A binding analysis. Briefly, HUVECs were de-attached using Accutase^TM^ cell detachment solution (BD Biosciences). HUVECs were washed with PBS and incubated with biotinylated recombinant VEGF-A or biotin control (biotinylated soybean trypsin inhibitor) in the absence or presence of MFB for 1 h. Cells were subsequently incubated with avidin-conjugated fluorescein for another 30 min. Cells were washed with PBS and fluorescence derived from labeled cells was determined by flow-cytometry and analyzed by CellQuest software (FACScan; BD Biosciences, San Jose, CA, USA).

***Molecular docking simulation***

For docking simulation, the X‐ray crystallography structure for VEGF‐C (PDB ID: 4BSK) (Leppanen et al., 2013) was obtained from RCSB Protein Data Bank. The preparation of protein was performed by Prepare Protein module in Discovery Studio 2.5 (DS2.5) to remove crystal water in crystallography structure, insert missing atoms in incomplete residues, protonate the structure of both proteins with Chemistry at Harvard Macromolecular Mechanics (CHARMM) force field (Brooks, Bruccoleri, Olafson, States, Swaminathan & Karplus, 1983), and optimize side‐chain conformation for residues with inserted atoms. For VEGF-C, we define the binding site at position (x,y,z) = (-74.24, 16.87, -13.50) with the volume of 367.750 Å^3^. The binding site of VEGFR-3 was defined as volume of the co-crystallized compound in the X-ray crystallography. Ligand Fit module in DS2.5 was performed to obtain the docking poses of compound using a shape filter and Monte‐Carlo ligand conformation generation and optionally minimized with CHARMM force field (Brooks, Bruccoleri, Olafson, States, Swaminathan & Karplus, 1983).

***Cell viability assay***

Colorimetric MTT assay was used to determine cell viability as described previously (Lien et al., 2019).

***Tail bleeding time analysis***

Mice used in the Matrigel plug assay were also employed to examine the effects of MFB on tail bleeding time. After matrigel implantation, animals were randomized to either the vehicle-treated control group or the MFB-treated group. Mice were intraperitoneally administrated with MFB once daily for 10 days. At the end of treatment, the mouse was placed in a tube holder with its tail protruding and 2 mm segment from the distal tail was severed. The amputated tail was immediately immersed in isotonic saline at 37℃. Bleeding time was recorded for a maximum of 1800 s and the end point was the arrest of bleeding (Lien et al., 2019).

**Supplement Figures**


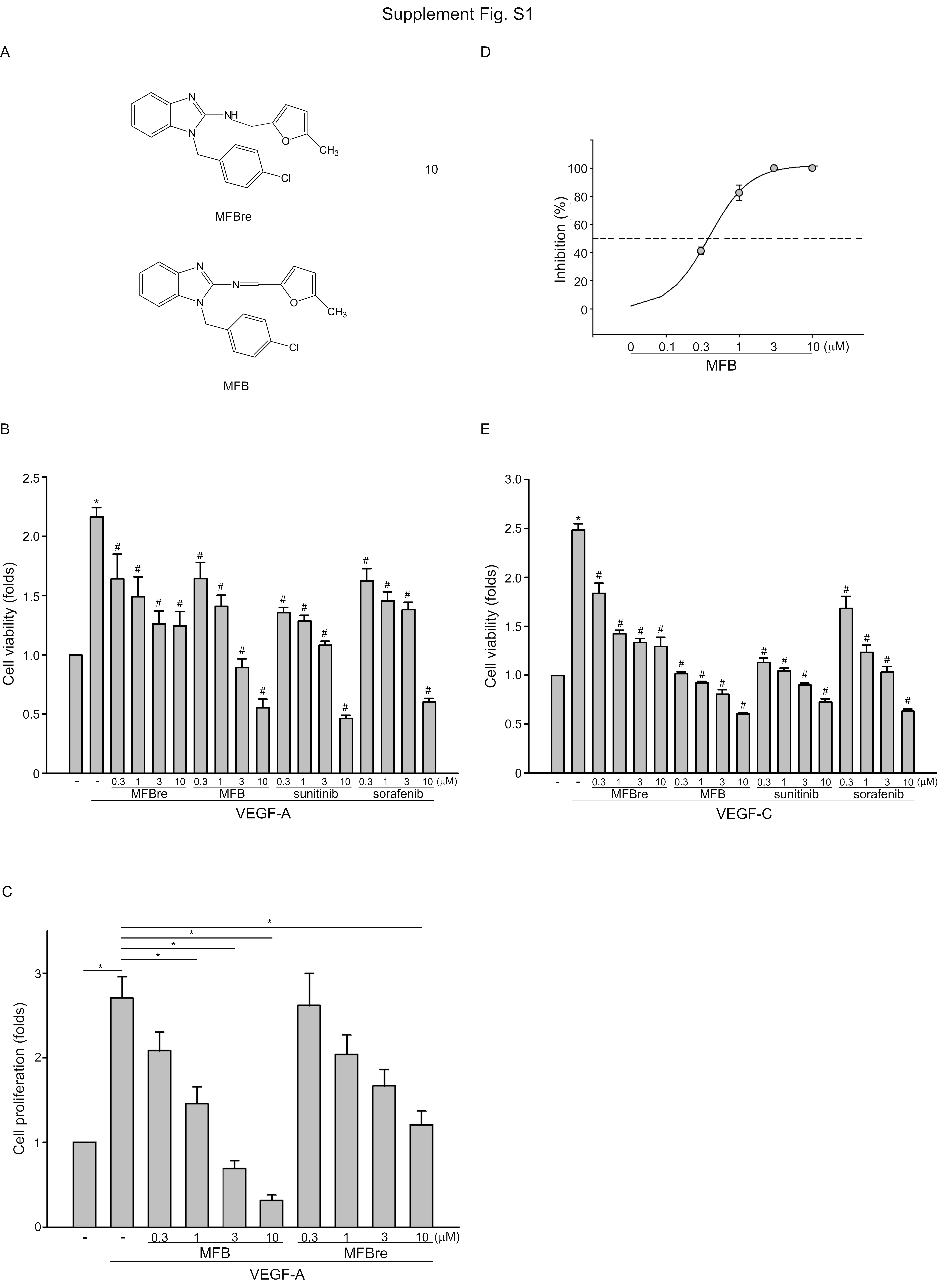
**Supplement Figure S1. Effects of MFB and MFBre on VEGF-A-induced cell proliferation in HUVECs** (A) Chemical structures of MFBre and MFB. (B) HUVECs were starved in 2 % FBS-containing M199 without ECGS for 18 h. After starvation, cells were treated with MFBre, MFB, sunitinib or sorafenib, followed by the stimulation with VEGF-A (25 ng/ml) for another 24 h. Cell viability was determined by MTT assay. Each column represents the mean ± S.E.M. of eight independent experiments performed in duplicate. *P < .05, significantly different from vehicle-treated control group; #P < .05, significantly different from the group treated with VEGF‐A alone (C) After starvation and treatment as described in (B), cell proliferation was determined by a BrdU-based cell proliferation assay. Each column represents the mean ± S.E.M. of eight independent experiments performed in duplicate. *P < .05, significantly different from the group treated with VEGF‐A alone (D) Based on the results derived from (C), the percentage of inhibitory effects of MFB on VEGF-A-induced HUVEC proliferation was calculated. (E) SV-LECs were starved in serum-free DMEM for 18 h. After starvation, cells were treated with MFBre, MFB, sunitinib or sorafenib, followed by the stimulation with VEGF-C (100 ng/ml) for another 24 h. Cell viability was determined by MTT assay. Each column represents the mean ± S.E.M. of eight independent experiments performed in duplicate. *P < .05, significantly different from vehicle-treated control group; #P < .05, significantly different from the group treated with VEGF‐C alone

**
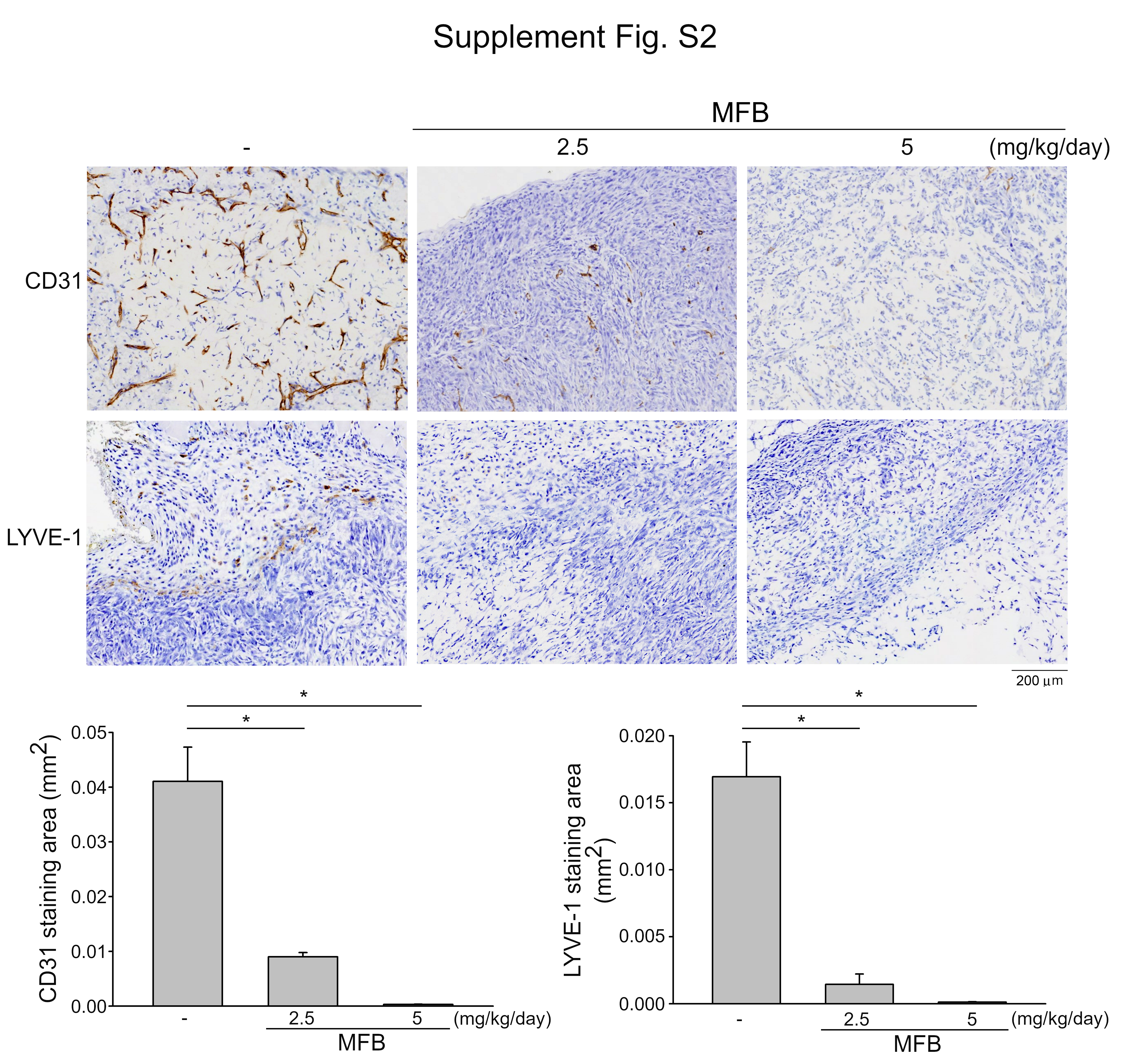
**

**Supplement Figure S2. Effects of MFB on tumor cells-induced microvessel and lymphatic vessel formation *in vivo*.** Matrigel mix with GBM8901 cells was subcutaneously injected into the right flank of nude mice. After implantation, mice were administrated intraperitoneally with vehicle or MFB for 10 days. Matrigel plugs removed from the mice treated with vehicle or MFB were subjected to immunohistochemical analysis to determine the effects of MFB on tumor cells-induced microvessel (CD31^+^) and lymphatic vessel (LYVE-1^+^) formation as described in the “Supplement Methods” section. Each column represents the mean ± S.E.M. of six plugs in each group *P < .05, significantly different from the vehicle-treated group; one‐way ANOVA, with Tukey's post‐hoc test.

**
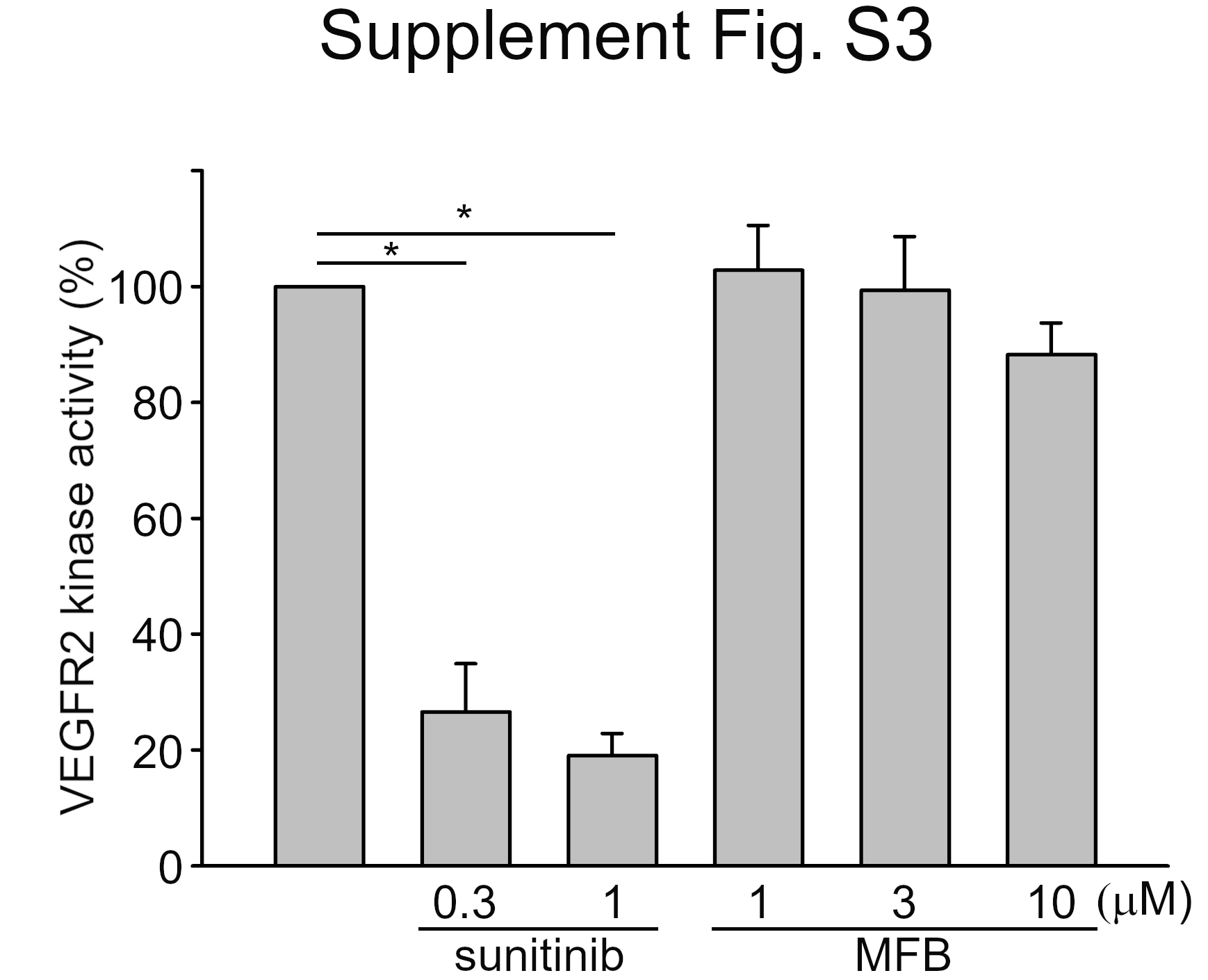
**

**Supplement Figure S3. Effects of MFB and sunitinib on VEGFR-2 kinase activities** Effects of MFB and sunitinib on VEGFR-2 kinase activity were determined by *in vitro* kinase assay using Kinase-Glo Plus luminescence kinase assay kit (Promega, Madison, WI, U.S.A.) as described in the “Supplement Methods” section. Each column represents the mean ± S.E.M. of five independent experiments. *P < .05, significantly different from control group.

**
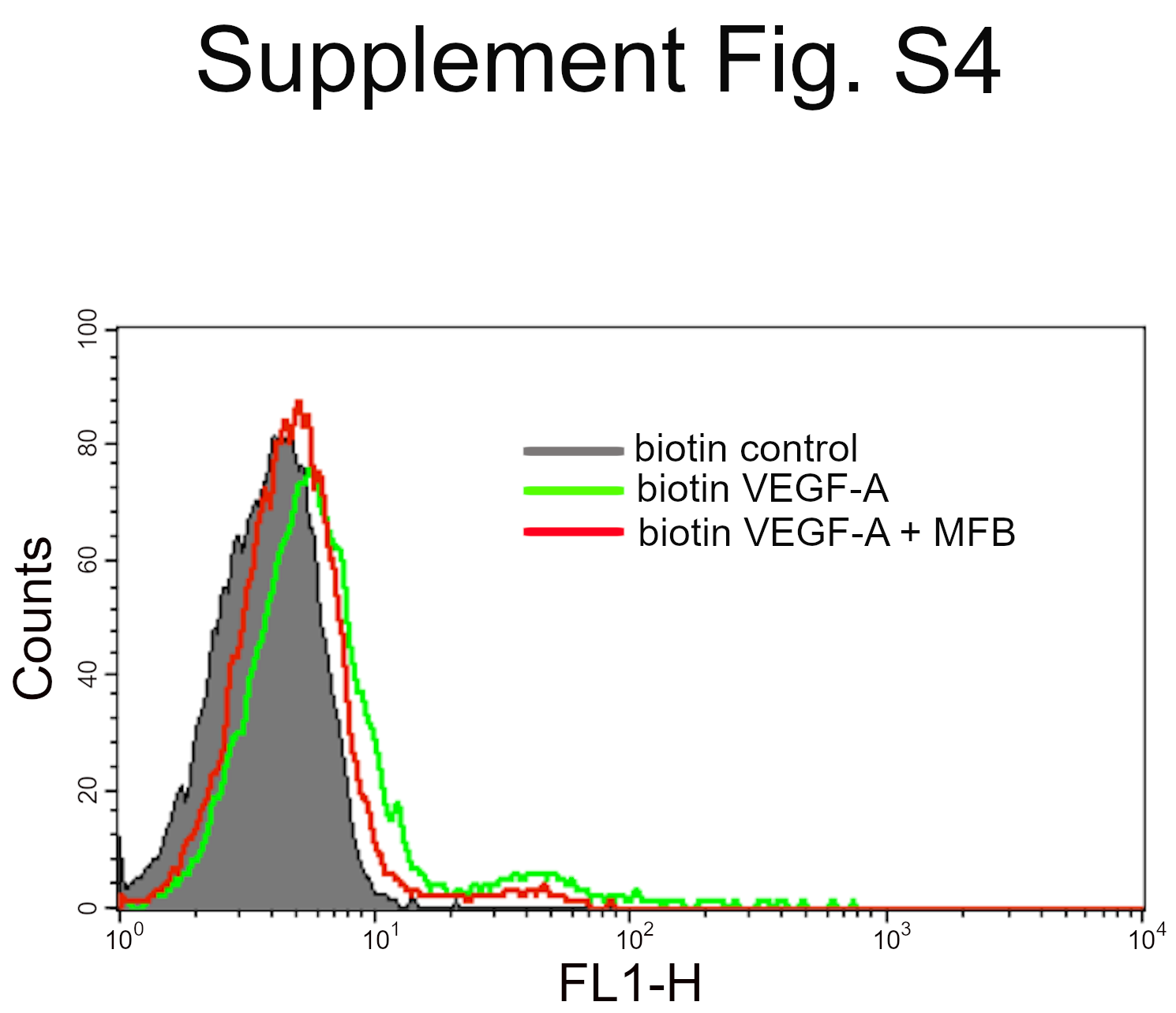
**

**Supplement Figure S4. VEGF-A binding assay of MFB.** HUVECs were detached, suspended in PBS and treated with biotin control or biotin VEGF-A in the absence or presence of MFB. After treatment, cells were treated with fluorescein-avidin and the fluorescence derived from biotin VEGF-A-stained cells were examined by flow-cytometry as described in the “Supplement Methods” section. Results shown are representative of three independent experiments.

**
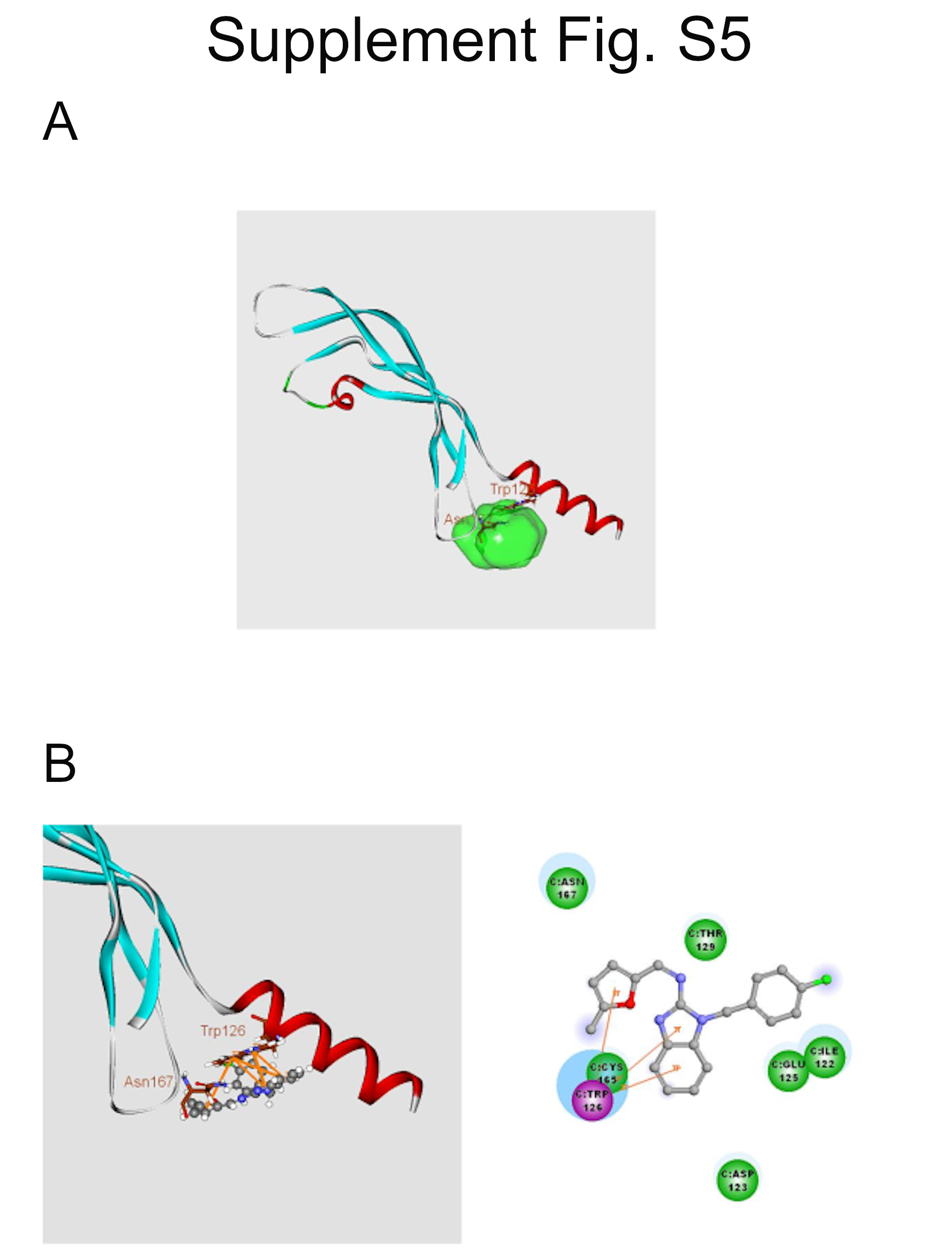
**

**Supplement Figure S5. The predicted binding models of MFB and VEGF-C.** (A) The binding site of VEGF-C was defined by two small receptor cavities between VEGF-C and VEGFR-3. Green, the putative binding site; Blue and Red, VEGF-C protein. (B) The possible docking pose of VEGF-C with MFB (left panel) and their 2D ligand-protein interaction diagram (right panel).

**
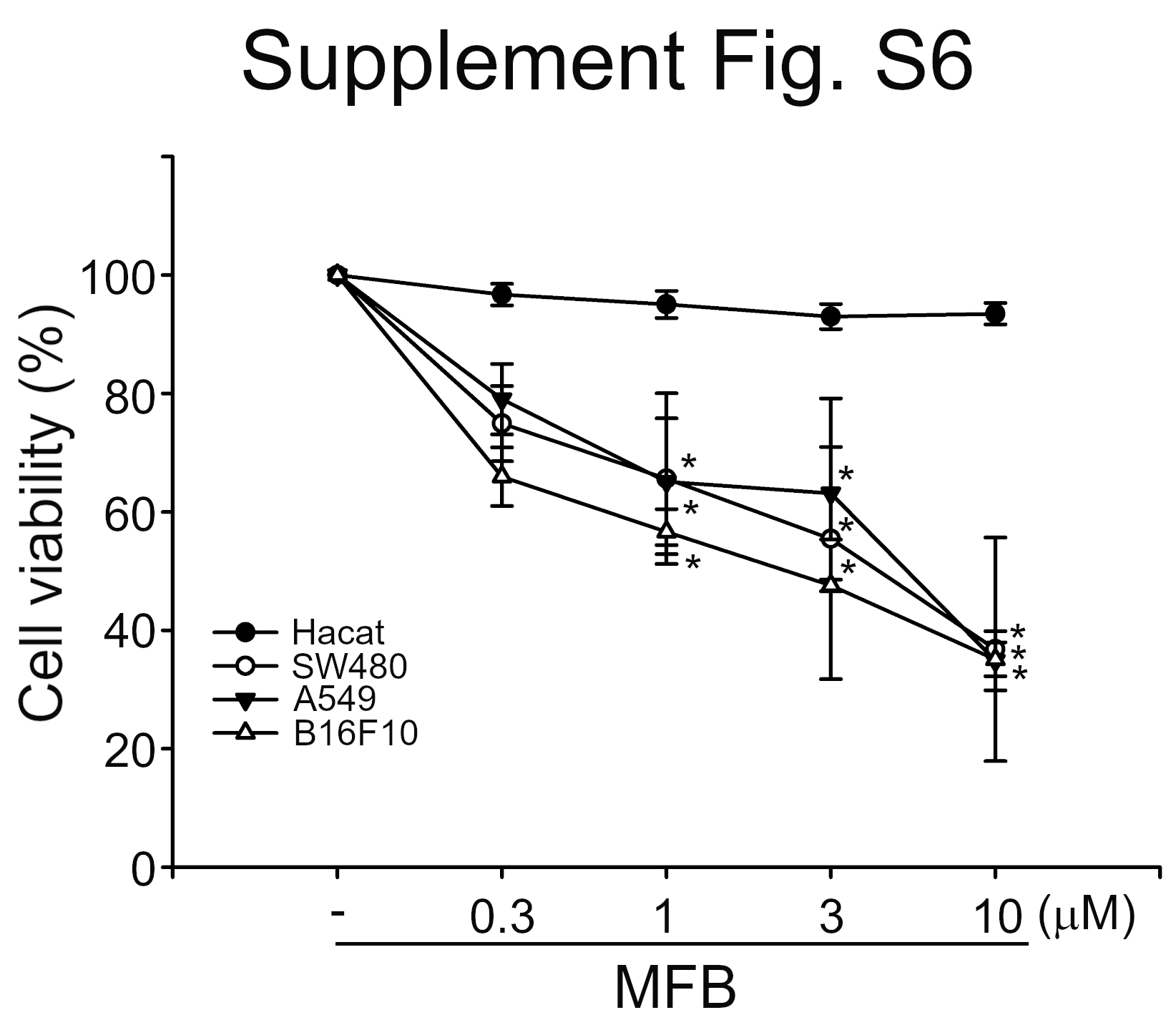
**

**Supplement Figure S6. Effects of MFB on cell viability in tumor cells or non-tumor Hacat keratinocytes.** Cells were treated with MFB at the concentrations of 0.3, 1, 3, or 10 μM for 24 h. Cell viability was determined by MTT assay. Each column represents the mean ± S.E.M. of eight independent experiments performed in duplicate. **p* < 0.05, compared with the control group.

**
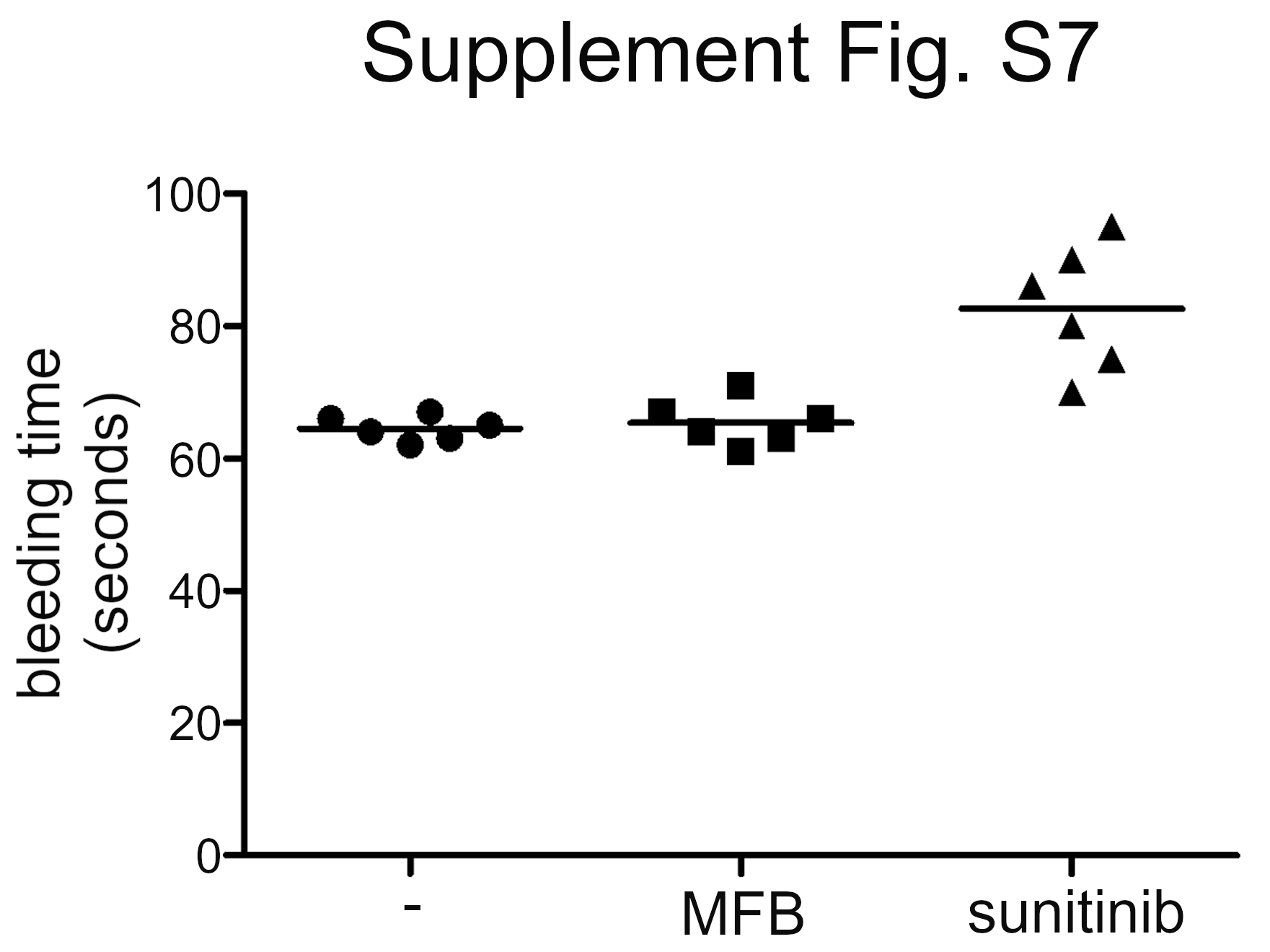
**

**Supplement Figure S7. Effects of MFB on tail bleeding times of mice.** Mice were intraperitoneally administrated with vehicle, MFB (5 mg/kg/day) or sunitinib (5 mg/kg/day) for 10 days. Tail bleeding time was determined. Each column represents the mean ± S.E.M. (N=6 for each group).

**
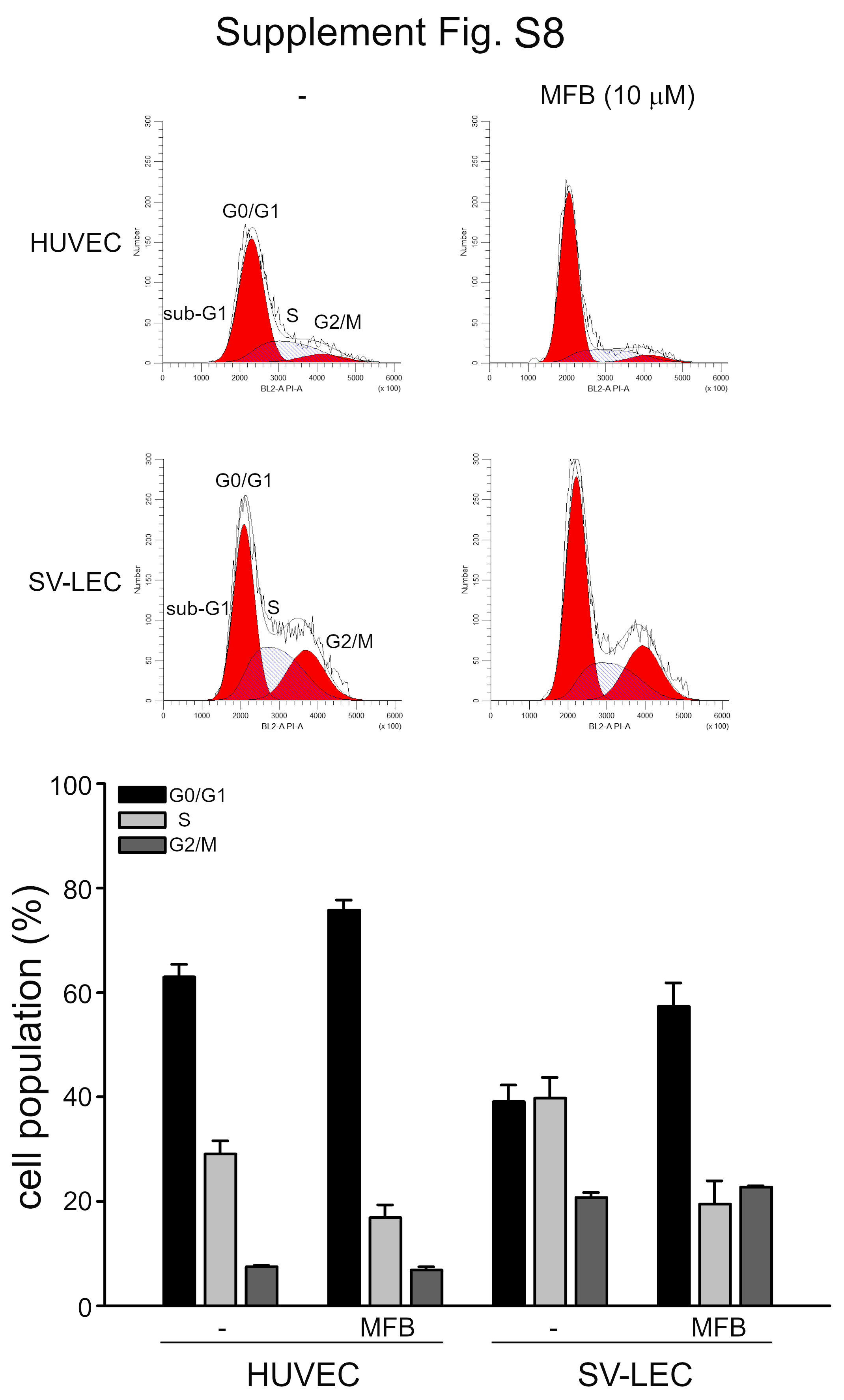
**

**Supplement Figure S8. Effects of MFB on cell cycle distribution in HUVECs or SV-LECs.** HUVECs or SV-LECs were treated with vehicle or MFB (10μM) for 24 h. The percentage of propidium iodide-stained cells in sub-G1 (apoptosis), G0/G1, S, and G2/M phases were analyzed by flow-cytometry as described in the “Supplement Methods*”* section. Each column represents the mean ± S.E.M. of three independent experiments

**Table S1. Scoring functions of MFB with VEGF-C**

| Protein | Compound | Pose | -PLP1 | -PLP2 | -PMF |
| --- | --- | --- | --- | --- | --- |
| VEGF-C | MFB | 1 | 72.43 | 71.25 | 85.44 |

Triple consensus scoring: PLP1, PLP2, and PMF. Piecewise Linear Potential (PLP); Potential of Mean Force (PMF).

**References:**

Brooks BR, Bruccoleri RE, Olafson BD, States DJ, Swaminathan S, & Karplus M (1983). CHARMM: A program for macromolecular energy minimization and dynamics calculations. Journal of Computational Chemistry 4**:** 187–217.

Leppanen VM, Tvorogov D, Kisko K, Prota AE, Jeltsch M, Anisimov A*, et al.* (2013). Structural and mechanistic insights into VEGF receptor 3 ligand binding and activation. Proc Natl Acad Sci U S A 110**:** 12960-12965.

Lien JC, Chung CL, Huang TF, Chang TC, Chen KC, Gao GY*, et al.* (2019). A novel 2-aminobenzimidazole-based compound Jzu 17 exhibits anti-angiogenesis effects by targeting VEGFR-2 signalling. Br J Pharmacol 176**:** 4034-4049.
